# How straw returning impacts microbial-interaction-network-mediated improvements in soil multifunctionality

**DOI:** 10.3389/fmicb.2025.1710232

**Published:** 2025-12-10

**Authors:** Haogeng Zhao, Zhanyuan Lu, Meijuan Cheng, Shuli Wei, Jing Fang, Wenqing Cao, Huimin Shi, Wei Zhang, Xiangqian Zhang, Yan Qu, Lingyue Liu, Dejian Zhang, Xiaoqing Zhao

**Affiliations:** 1 School of Life Science, Inner Mongolia University, Hohhot, China; 2 Inner Mongolia Academy of Agricultural and Animal Husbandry Sciences/Key Laboratory of Black Soil Protection and Utilization/Inner Mongolia Key Laboratory of Degradation Farmland Ecological Restoration and Pollution Control, Hohhot, China; 3 College of Agronomy, Inner Mongolia Agricultural University, Hohhot, China; 4 Arong Banner Agricultural Technology Extension Center, Hulun Buir, China

**Keywords:** black soil, straw returning, rhizosphere microbial community, soil multifunctionality, co-occurrence network, production capacity improvement

## Abstract

**Introduction:**

Straw returning is an important agricultural measure for improving soil health; however, the mechanism driving how it shapes the characteristics and multifunctionality of crop rhizosphere microbial communities in black-soil areas remains unclear.

**Methods:**

Through a 3-year field experiment, combined with high-throughput sequencing, co-occurrence network analysis, and multi-model coupling, this study systematically analyzed the effects of different straw-returning rates (no straw returning, half-dose straw returning, and full-dose straw returning) on the characteristics and multifunctionality of crop rhizosphere soil microbial communities in typical black-soil areas.

**Results:**

The results showed that the half-dose straw-returning treatment significantly increased soil microbial diversity, whereas the full-dose straw-returning treatment significantly enriched bacterial groups such as o_Rokubacteriales and f_Anaerolineaceae and fungal groups such as Ophiocordyceps and Gigaspora. Soil nitrate nitrogen, organic carbon, microbial biomass nitrogen, microbial biomass phosphorus, and invertase activity were important factors affecting soil multifunctionality. Furthermore, co-occurrence network analysis indicated that the nodes, edges, and average degree of the fungal community under the half-dose straw-returning treatment significantly increased by 9.41, 15.93, and 30.13%, respectively, compared with those in the no-straw-returning treatment. There was a significant positive correlation between the complexity of the fungal network and microbial network and the soil multifunctional index. Additionally, microbial network complexity is a direct key factor that drives improvements in soil multifunctionality, and microbial diversity indirectly enhances soil multifunctionality by regulating microbial network complexity. Moreover, soil multifunctionality was significantly positively correlated with crop biomass and grain yield.

**Discussion:**

This study elucidates the mechanism by which straw returning enhances black-soil fertility through the regulation of soil microbial networks, providing a theoretical basis and practical value for sustainable agricultural development and straw resource utilization in black-soil regions.

## 1 Introduction

Black soil is a type of soil characterized by its high organic matter content and exceptional fertility, and the quality of black soil and its ecological functions are closely associated with sustainable agricultural development (Liu et al., 2019). In recent years, black soil has suffered losses in organic matter, impaired nutrient retention capacity, and significant changes to microbial communities due to increased production with unsound fertilization practices. Such degradation is a major threat to soil health in black-soil regions and reduces the yield and quality of crops (Dan et al., 2008; Sun et al., 2016). As a source of exogenous organic matter input, straw returning has been identified as being, for the most part, an important strategy to improve soil structure and fertility (Zhao et al., 2016). For example, straw returning is known to increase organic matter content in soil when compared to only applying chemical fertilizers (He et al., 2015). Organic matter content increases soil’s nutrient supply capacity and improves its physical structure and biological properties (Yan et al., 2022; Yang et al., 2012). Soil microorganisms are the main drivers of biogeochemical cycles, and their community structures and functions are closely related to organic matter content (Yan et al., 2022). The products of straw degradation can drive dynamic changes in soil fertility by regulating the diversity of soil microbes and ensuring the stability of their community structure, creating a virtuous cycle primarily encompassing soil microorganisms, organic matter, and straw degradation (Yan et al., 2022).

Soil multifunctionality is the capacity of soil to deliver and sustain multiple ecosystem functions simultaneously, such as microbial activity, nutrient cycling, soil nutrient storage, soil nitrogen transformations, and litter breakdown. Soil multifunctionality mainly concerns the overall performance of soil in terms of various ecosystem functions, as opposed to the study of single or multiple soil functions (Manning et al., 2018; Zhao and Wang, 2024). The interaction between soil multifunctionality and soil microbial diversity has become a research hotspot (Zheng et al., 2019). While many studies have identified a strong association between soil microbial diversity and soil multifunctionality (Delgado-Baquerizo et al., 2016b; Wagg et al., 2019; Zhang et al., 2021) and found that high diversity is needed to support multifunctionality, some studies have also shown that the two are not related (Wagg et al., 2014) or are even negatively correlated (Wang et al., 2021). This has resulted in a great deal of disagreement concerning the relationship between soil microbial diversity and ecological function. The main reason for this is that the initial studies concentrated on the relationships between a single ecological function and the number of species in the context of microbial diversity, without recognizing the complex role microbial populations can play in supporting ecological functions (Ma et al., 2024). In natural ecosystems, microorganisms form a complex microbial interaction network through various direct or indirect ecological interactions, such as synergistic symbiosis, resource competition, and antagonism. This network structure is an important factor in maintaining the basic ecological functions of an ecosystem, such as energy flow, material circulation, and information transmission (Zhang J. et al., 2022). Research on the management of indoor microbial diversity indicates that the complexity of the soil microbial network affects ecosystem functions, with possible consequences that exceed a microbial community’s species diversity (Wagg et al., 2019). Previous studies of straw returning in black-soil areas mainly examined the temporal and spatial heterogeneity of the physical and chemical properties and enzyme activities of non-rhizosphere soil as well as changes in microbial community diversity and the composition of the community structure across vertical profiles (Guan et al., 2023; Zhao et al., 2016). Nevertheless, there has been insufficient research on the relationship between microbial structural characteristics and soil multifunctionality in crop rhizosphere soil at different straw-returning rates.

A soybean field located in a black-soil area was selected as the research site. After 3 years of positioning testing, high-throughput sequencing, co-occurrence network analysis, and multi-model coupling, changing the amount of straw returned to the soil (0, 50, and 100%), analyses of rhizosphere soil microbial diversity, community structure composition, and network complexity were conducted systematically. The microbial factors that contribute to changes in soil multifunctionality were clarified, and the relationships between soil multifunctionality and the soil’s bacterial, fungal, and microbial communities were determined. The objectives of the research were to (1) clarify the changes in rhizosphere soil microbial community and soil multifunctionality under different soybean straw-returning treatments, (2) identify the microbial drivers of soil multifunctionality, and (3) clarify how the microbial diversity–community structure composition–network complexity–multifunctionality cascade regulation mechanism enhances the productivity of crops. The findings provide a theoretical basis for efforts to analyse the microbial mechanisms driving improved soil function under agricultural management measures and have important implications for soil health regulation and the utilization of straw resources in black-soil regions.

## 2 Materials and methods

### 2.1 Study area

The test site was located in the long-term positioning observation station of soil management and ecological restoration in Arong Banner, Inner Mongolia Academy of Agricultural and Animal Husbandry Sciences (47°56′–49°19′N, 122°2′–124°5′E). It is located in Arong Banner County, Hulunbuir City, Inner Mongolia Autonomous Region. The region is characterized as a temperate continental monsoon climate zone. The soil type is black soil, the average annual temperature is 1.7 °C, the frost-free period is 90–130 days and the average annual precipitation is 458.4 mm. June–August precipitation contributes approximately ~70% annual precipitation, while annual sunshine duration is 2,750–2,850 h. At the beginning of the experiment in 2022, 10 sampling points were randomly selected within the experimental area, and soil samples from 0 to 20 cm depth were collected. The average of the measurements from these 10 sampling points was used to represent the baseline nutrient status before the experiment. The soil pH was 7.8, soil organic carbon (SOC) of 19.2 g kg^−1^, total nitrogen (TN) of 1.4 g kg^−1^, total phosphorus (TP) of 1.04 g kg^-l^, available phosphorus (AP) of 7.9 mg kg^−1^ and available potassium (AK) of 30.2 mg kg ^−1^.

### 2.2 Experimental design

The long-term positioning experiment was initiated in 2022, and the crop planted was soybean, with one cropping cycle per year. The experiment was designed with three straw-returning treatments: (1) no straw returning (CK), (2) half-dose straw returning (SBSH), and (3) full-dose straw returning (SBSF). Each treatment had three replications, and the plot area was 100 m^2^ (20 m × 5 m). The straw return treatments were initiated in 2022. Straw is returned to the field annually, and this is performed only after crop harvesting. Under the no-straw-returning treatment, all of the straw was completely removed from the plot, and half of the amount was returned to the field. This was achieved by manually removing 50% of the straw of the aboveground part of the plot, ensuring that the remaining part uniformly covered the plot after mechanical crushing, and then incorporating it into the 0–30 cm soil layer by plowing. With respect to SBSF, all of the straw was crushed and returned to the field via a mechanical method and similarly incorporated into the 0–30 cm soil layer via plowing. The straw return rate was 4,200 kg/hm^2^ for the full-dose straw returning (SBSF) treatment and 2,100 kg/hm^2^ for the half-dose straw returning (SBSH) treatment. The planting density of the soybean was 300,000 plants/hm^2^. The following fertilizers were incorporated as basal dressing concurrently with sowing at 50, 150, and 50 kg hm^−2^: urea (N content, 460 g kg^−1^), diammonium phosphate (N content, 180 g kg^−1^; P content, 460 g kg^−1^), and potassium sulfate (K_2_O content, 500 g kg^−1^), respectively.

### 2.3 Sample collection

The flowering stage represents a period of peak root exudation and microbial activity, driven by the high nutrient demand associated with pod formation. Sampling at this stage makes it possible to capture treatment-induced differences when microbial community structure and function exhibit the greatest divergence and are most closely correlated with yield formation. Accordingly, from 2022 to 2024, rhizosphere soil samples were collected annually in July during the flowering stage of soybean plants for systematic analysis of soil physicochemical properties. After significant differences in both soybean phenotypes and soil characteristics were confirmed across treatments, microbial community analysis was conducted on samples collected in July 2024. Ten uniformly growing plants were selected from each plot. Their roots were carefully excavated, and loosely adhering soil was removed. Rhizosphere soil (within 0–1 mm from the root surface) was collected with a sterile brush and combined into a composite sample. This process yielded a total of three soil samples per treatment. The fresh soil was then sieved through a 2 mm mesh to remove visible impurities. The sieved soil was divided into three aliquots: one was air-dried for physicochemical analysis, another was stored at 4 °C for the measurement of microbial biomass and enzyme activities, and the third was preserved at −80 °C for subsequent microbial sequencing.

The plant samples corresponding to the collected rhizosphere soil were used to determine soybean plant height and fresh weight, with measurements taken from the stem section between the base and the apex. Plant height was measured as the vertical length of this segment, while fresh weight was determined as the actual biomass of the same plant part. Grain yield was assessed at maturity by selecting the central four rows in each plot and harvesting a 2-meter section from each row, with three replicates. The sampling area per replicate was 4.8 m^2^, and the yield was converted to a standardized unit area yield.

### 2.4 Soil physiochemical analysis

The soil organic carbon (SOC) content was calculated by the dichromate oxidation method (Mebius, 1960). The total nitrogen (TN) content was quantified following methods for Kjeldahl digestion (Bremmer and Mulvaney, 1996). The soil nitrate (NO_3_^−^-N) and ammonium (NH_4_^+^-N) contents were analyzed via extraction with a 1 mol L^−1^ KCl solution with a sample-to-solution ratio of 1:10 w/v, and analyzed directly via a continuous flow injection analyzer (FLOWSYS, Italy). The total phosphorus (TP) content was analyzed via sulfuric acid-perchloric acid digestion, then analyzed using the ascorbic acid-molybdophosphate blue method (Pascual et al., 2015). The available phosphorus (AP) content was quantified via sodium bicarbonate (NaHCO_3_) extraction method (Olsen, 1954). The soil microbial biomass carbon (MBC), nitrogen (MBN), and phosphorus (MBP) contents were estimated using the chloroform fumigatio-extraction method (Brookes et al., 1985; Vance et al., 1987). The activities of the major soil carbon (soil sucrase, SSC), nitrogen (soil urease, Ure), and phosphorus (soil alkaline phosphatase, ALP) cycling enzymes were quantified colorimetrically using soil enzyme activity assay kits (Beijing Solarbio Science and Technology Co., Ltd.; BC0245, BC0125, and BC0285, respectively).

### 2.5 DNA extractions, sequencing, and data processing

DNA was extracted from rhizosphere soil samples following the protocol of FastDNA™ SPIN Kit for Soil (MP Biomedicals, United States) and the concentration and purity of the DNA was measured using NanoDrop One. The V4 and V5 regions of soil bacterial 16S rRNA genes were amplified by polymerase chain reaction (PCR) with the primers 515F (5′-GTGCCAGCMGCCGCGGTAA-3′) and 806R (5′-GGACTACHVGGGTWTCTAAT-3′). Fungi were amplified by PCR using ITS1F (5′-CTTGGTCATTTAGAGGAAGTAA-3′) and ITS2R (5′-GCTGCGTTCTTCATCGATGC-3′). Amplification PCR products of soil sam ples were visible by 2% agarose gel electrophoresis and target fragments were extracted using an E. Z. N. A. Gel Extraction Kit (Omega, United States) gel recovery kit (Behnke et al., 2011; Gardes and Bruns, 1993). The target band gel was extracted and purified to construct the library and high-throughput sequencing was performed on the Illumina MiSeqPE250 platform (Majorbio Bio-pharm Technology Co., Ltd., Shanghai, China) (Han et al., 2024). The raw sequencing data were spliced after low-quality bases and primer adaptor sequences were removed, and the sequences that were shorter than 160 bp were eliminated. The effective sequence data of each sample were obtained following the removal of nonspecific amplification sequences and chimeras. The 16S and ITS sequences were assigned to operational taxonomic units (ASVs) 97% similarity threshold. Follo wing QIIME, the percentage identity of families was calculate using UCLUST sequence comparison software that allows for merging per operational taxonomic unit (ASV) based on 97% sequence similarity, then the sequence with the highest sequence identity of each ASV was designated as the representative ASV sequence (Edgar, 2013). For bacterial 16S rRNA and fungal ITS rRNA, Greengenes and Silva databases were used as template sequences for OTU classification status identification (Wang et al., 2007). To reduce the influence of different sequencing depths between samples, the ASV abundance data were leveled to the minimum sample sequence depth, and 8,896 bacterial ASVs and 1939 fungal ASVs were obtained.

### 2.6 Calculation of soil multifunctionality

Twelve key ecosystem function indexes were selected to characterize soil multifunctionality (Luo et al., 2023; Wang X. et al., 2023). These indexes are closely related to the storage and circulation of carbon, nitrogen and phosphorus, which can well reflect the functions of ecosystem nutrient storage and utilization, soil fertility and biogeochemical cycle (Xiao Y. et al., 2023). Three methods are used to calculate the versatility index (Luo et al., 2023). First is the single-function index: multiple single functions represent the actual strength of versatility in each indicator, which is obtained by standardizing each directly measured indicator (the standardized value range is 0–1) (Wang X. et al., 2023). Second is the average multifunctionality index (AMI): this index is obtained by averaging the standardized values of each function and is the most widely used and easily calculated multifunctionality index (Delgado-Baquerizo et al., 2016a). Third is principal coordinate multifunctionality index (PMI): this index characterizes different dimensions of multifunctionality by extracting multiple principal axes from the principal coordinate analysis (PCoA) of all single functions (Ma et al., 2024; Manning et al., 2018). The principal coordinate versatility index uses the R package “vegan” to extract the first five principal axes based on the Bray–Curti’s distance to characterize different dimensions of multifunction, which can effectively prevent the overexpression of a single function.

### 2.7 Soil microbial diversity and community structures

A phylogenetic tree was built using the “metacoder” package and species richness and the phylogenetic diversity index were calculated using the “vegan” and “picante” packages. The richness values of bacteria and fungi were standardized (Formula 1), and the standardized scores were averaged to provide a microbial diversity index. The relative abundance of bacteria and fungi at the genus level in each sample was calculated with the “amplicon” package. The similarity of microbial community composition among samples was assessed via PCoA based on Bray–Curti’s distance. The effect of soybean straw return on microbial community was assessed using Permutational multivariate ANOVA (PerMANOVA, permutations = 999) (Anderson, 2014):


(1) $$\mathrm{STD}=\frac{\left(X-x_{\min}\right)}{\left(x_{\max}-x_{\min}\right)}$$

where *STD* is the standardized variable and *X*, *X_min_*, and *X_max_* are the target variables, minimum values and maximum values of all samples, respectively.

### 2.8 Construction and analysis of bacterial–fungal ecological networks

Using all rhizosphere soil samples for each treatment, bacterial-fungal cross-boundary symbiotic, bacterial, and fungal networks were constructed. Only ASVs with an average relative abundance higher than 0.5% in rhizosphere samples were kept to focus on the core microbial community and reduce noise from sparse data. Using the “psych” package, Spearman correlations were calculated for ASVs, and only nodes/edges with| *r*| > 0.70 and *p* < 0.01 significance positive edges (FDR adjusted for multiple testing) were extracted. The co-occurrence network was built using the “igraph” package, and visualization of the network was done using Gephi 0.0.1 software (Yi et al., 2023). The “igraph” package was used to extract the subnetworks of each sampling point from the three networks created above using the “subgraph” function. Also, the topological properties are calculated to investigate the characteristics of networks such as number of nodes and edges, average degree, clustering coefficient, average path length, network density and betweenness centrality (Qiu et al., 2021; Zhang et al., 2018). Subsequently, PcoA was conducted on the topology characteristics of the subnetwork to obtain indicators reflecting the complexity of the microbial network.

### 2.9 Data synthesis and statistical analysis

All data analyses were performed using R (version 4.4.1) (Team, 2020). One-way ANOVA was used to analyze soil microbial diversity, community structure and soil multifunctionality among treatments. The least significant difference method was used for the data analysis with a significance level of *p* = 0.05. Pearson correlation analysis was used to clarify the relationships between microbial diversity, network topology, and soil multifunctionality. Linear regression analysis was performed to investigate the relationship of microbial diversity, microbial community structure and microbial network complexity in relation to soil multifunctionality. FAPROTAX (Liang et al., 2020) was utilized to determine bacterial functions and FUN Guild (Nguyen et al., 2016) was used for fungi. A random forest (RF) model using the R software package “random Forest” was also conducted, in order to indicate the main drivers of multifunctionality, with the average importance of each of the developed variables represented by the percentage mean square error (Liaw and Wiener, 2002). A structural equation model (SEM) was constructed using the “piecewise SEM” package, in order to further investigate the direct and indirect relationships between microbial diversity, community structure composition, microbial network complexity and soil multifunctionality, and the subsequent effects of multifunctionality on crop growth and yield. The model fit was assessed using Fisher’s C statistics (Lefcheck, 2016).

## 3 Results

### 3.1 Changes in soil microbial diversity and multifunctionality under different soybean straw-returning treatments

**Figure 1:**
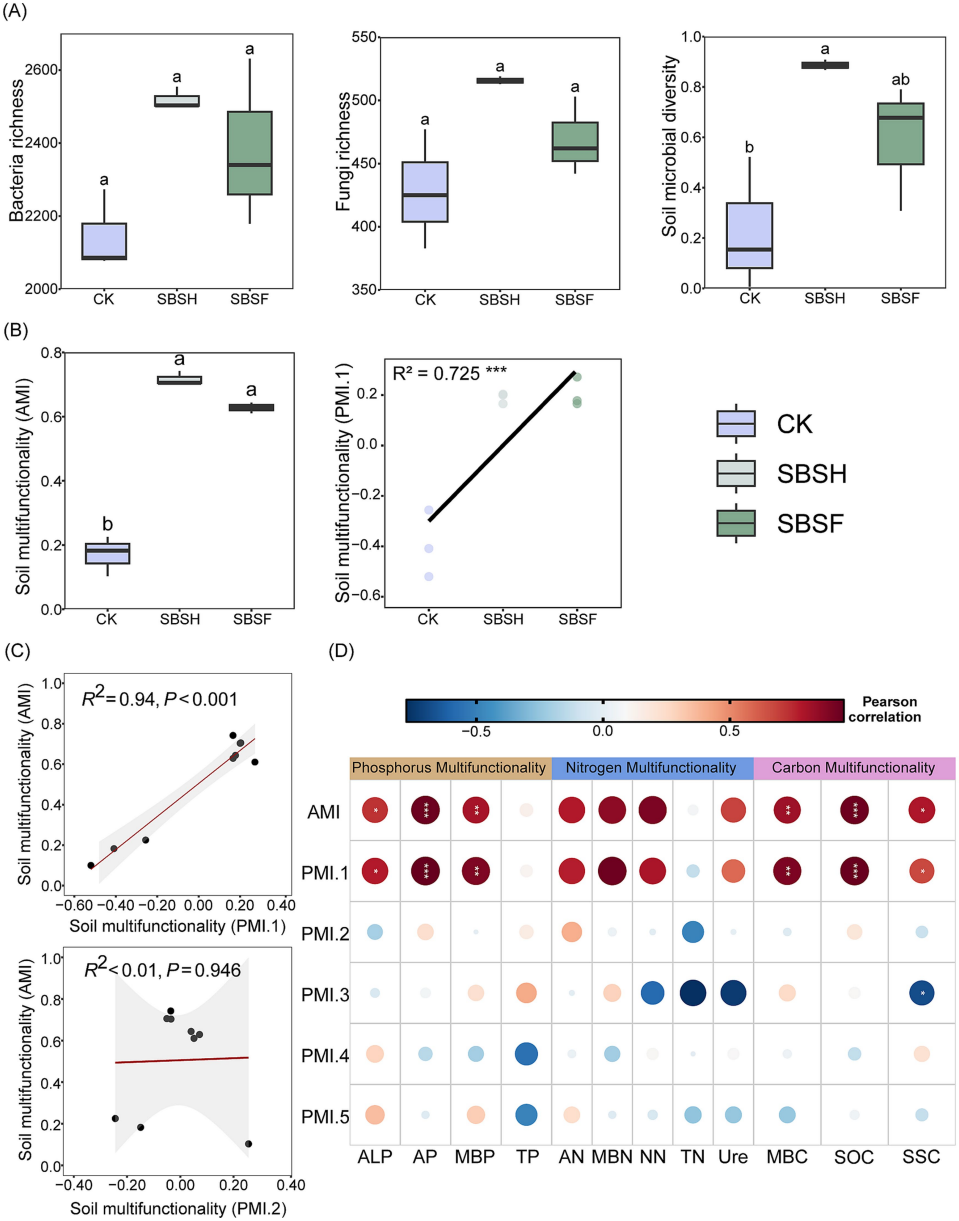
Soil microbial diversity index **(A)**. Multifunctionality index **(B)**, and the correlation between soil multifunctional indicators **(C,D)**. Different capital letters indicate significant differences in one-way ANOVA tests between different treatment groups (*p <* 0.05). PMI.1–PMI.5 represent the first five principal axes representing different dimensions of versatility extracted by the principal coordinate method. ALP, Alkaline phosphatase; AP, available phosphorus; TP, total phosphorus; MBP, soil microbial biomass phosphorus; AN, ammonium nitrogen; NN, nitrate nitrogen; MBN, soil microbial biomass nitrogen; TN, total nitrogen; Ure, urease; MBC, soil microbial biomass carbon; SOC, organic carbon; SSC, soil sucrase.

Species richness (reflected by the richness index) is used as a measure of soil microbial diversity; it is the broadest and simplest measure indicating changes in microbial diversity under different soybean straw-returning treatments. Soil microbial diversity was significantly different across the different treatments (*p* < 0.05). The SBSH and SBSF treatments showed no significant effect on the richness indices for bacteria and fungi compared to the CK treatment (*p* > 0.05). Soil microbial diversity was significantly greater and exhibited minimal variation under the SBSH treatment compared to the CK treatment (*p* < 0.05), indicating that this level of straw returning may not only improve but also stabilize the overall rhizosphere soil microbial community (Figure 1A). The average multifunctionality index (AMI) for the soybean straw-returning treatments (SBSH and SBSF) was statistically significantly increased (*p* > 0.05). Similarly, the analysis of the principal coordinate multifunctionality index (PMI.1) supported the notion that soybean straw returning significantly improved soil multifunctionality (*p* < 0.01). Moreover, functional improvements under the full-dose and half-dose returning treatments were similar (Figure 1B; Supplementary Figure S1).

The principal coordinate multifunctional indexes (PMI.1 and PMI.2), represented by the first two axes, provided by the PCoA and the soil AMI were used in the ordinary least squares linear regression analysis (Figure 1C). For increases in the PMI.1 principal coordinate multifunctionality index, there was a highly positive relationship with the soil AMI (*R*^2^ = 0.94, *p* < 0.001). In contrast, the PMI.2 principal coordinate multifunctionality index exhibited no relationship with the soil AMI (*R*^2^ < 0.01, *p* = 0.946). The analysis suggested there was a significant relationship between AMI and the single-function index (Figure 1D), except for TP and TN (*p* < 0.05), represented as the first axis in the principal coordinate index. However, for PMI.2-PMI.5, the single-function index showed almost no significant relationship (*p* > 0.05). Thus, PMI.1 was capable of demonstrating most of the functional variation in the soil. In the rest of the analysis, the single-function index-based PMI.1 would be retained.

### 3.2 Contribution of microbial diversity to the change of soil multifunctionality

**Figure 2:**
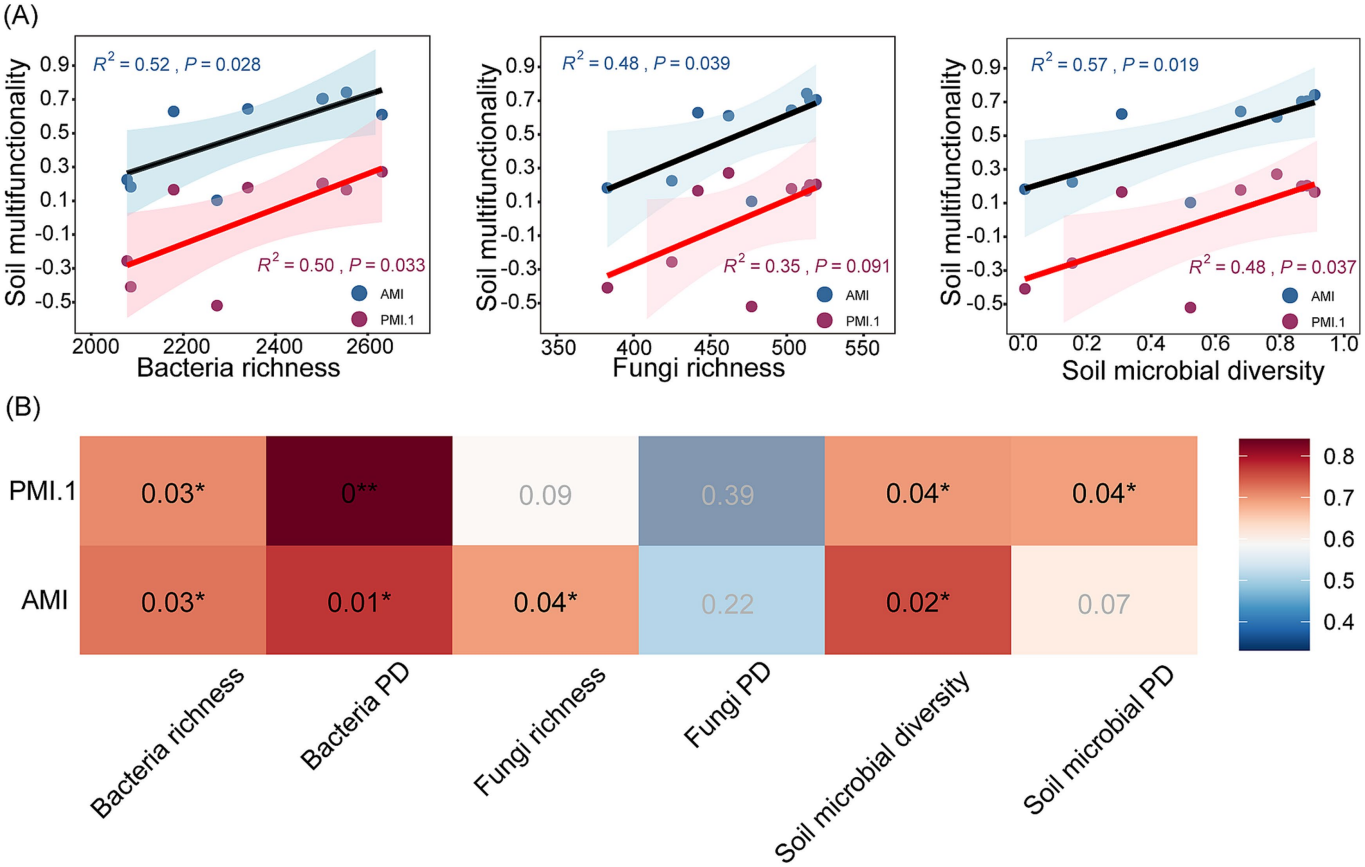
Linear regression **(A)** and correlation heat map **(B)** of soil microbial diversity and soil multifunctionality. In the linear regression, the blue and red scatter points represent AMI and PMI.1, respectively. The color in the heat map represents the Spearman correlation, and when the color is darker, the correlation is more significant. Significant results are indicated by *p < 0.05, **p < 0.01.

Based on the Spearman correlation analysis, bacteria/fungi richness, soil microbial diversity, and bacteria PD were significantly positively correlated with the AMI (*p* < 0.05). There was also a significant positive correlation between bacteria richness/PD, soil microbial diversity/PD, and PMI.1 (*p* < 0.05; Figure 2B). The effects of the bacterial diversity index and microbial diversity on the soil single-function index were greater than those of the fungal diversity index. The correlation between AP, AN, NN, SOC, SSC, and the diversity index was quasilinear (*p* < 0.05; Supplementary Figure S2). Through regression analysis, it was found that the regression variance coefficient (R^2^) of the fungal, bacterial, and microbial diversity indices in relation to the AMI was higher than that with respect to the principal coordinate multifunctional index (PMI.1; R^2^), indicating that the overall contribution of microbial diversity to soil multifunctionality (AMI) was significant, whereas the contribution to specific functional dimensions (PMI.1) was relatively weak. Moreover, it was found that the regression variance coefficient (R^2^) for microbial diversity and the AMI was higher than that of the fungal and bacterial richness indexes, indicating that microbial diversity as a whole had a strong ability to explain soil multifunctionality. By contrast, the regression variance coefficient between bacterial richness and the PMI (PMI.1) is larger, indicating that bacteria play a more prominent role in specific functional dimensions (PMI.1; Figure 2A).

### 3.3 Microbial community succession under different soybean straw-returning treatments

**Figure 3:**
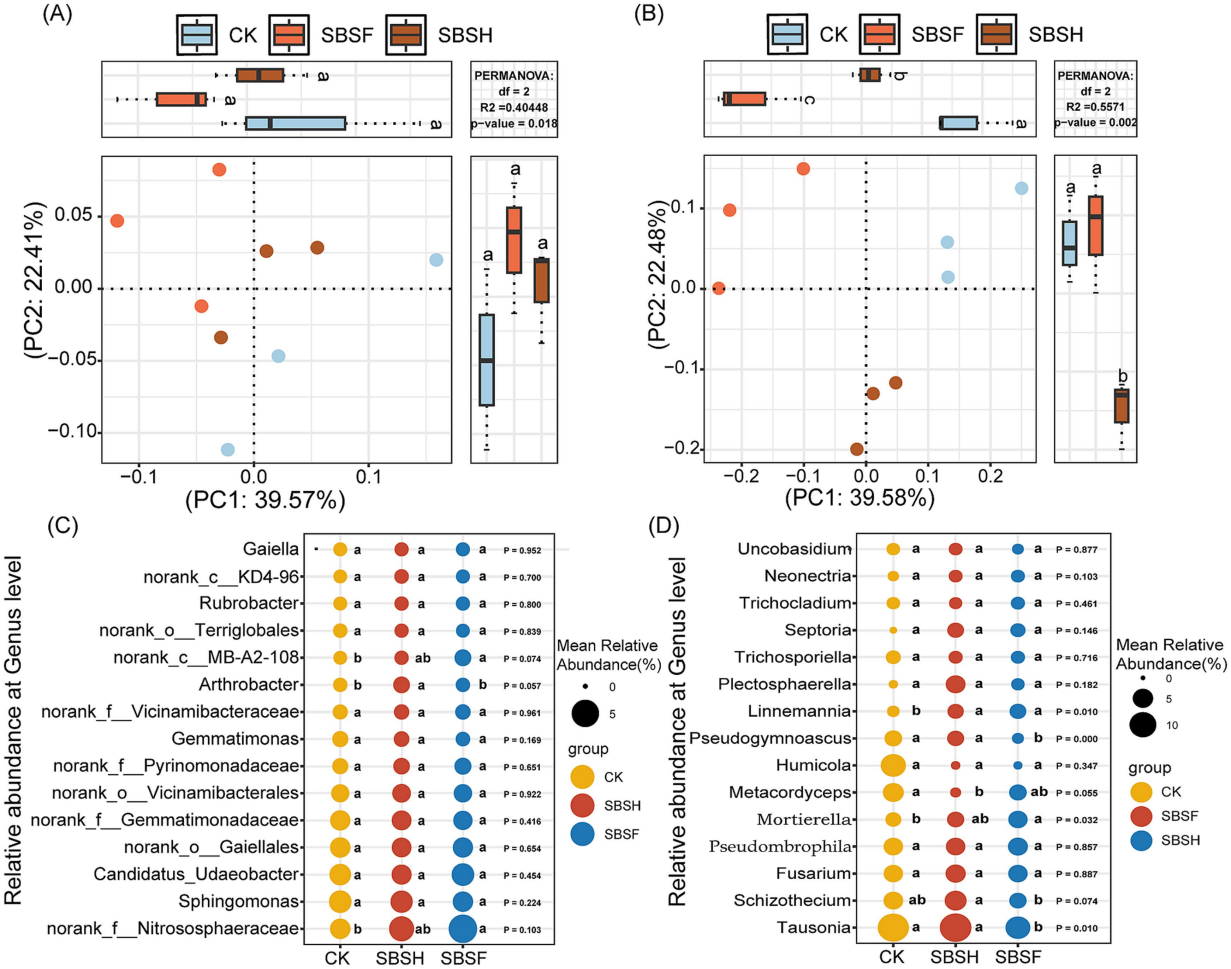
Permutational multivariate ANOVA **(A,B)** and microbial genus level composition **(C,D)** of soil microbial community under different straw returning amounts.

The results of the PCoA indicate that different soybean straw-returning treatments significantly impact the microbial community structure of rhizosphere soil. The composition of the genus-level bacterial community structure exhibited significant differences (*p* < 0.05), while the composition of the genus-level fungal community structure exhibited extremely significant differences (*p* < 0.01; Figures 3A,B). The microorganisms with relative abundances placing them in the top 15 bacteria and fungi were chosen for analysis. Relative to the CK treatment, the SBSF treatment significantly increased the relative abundances of *norank_f_Nitrososphaeraceae* and *norank_c_MB-A2-108,* by 124.67 and 76.14%, respectively. The SBSH treatment significantly increased the relative abundance of *Arthrobacter* by 52.41% (*p* < 0.05; Figure 3C; Supplementary Table S1). There were significant differences in the abundances of *Tausonia*, *Schizothecium*, *Mortierella*, *Metacordyceps*, *Pseudogymnoascus*, and *Linnemannia* in the fungal community (*p* < 0.05). The SBSF treatment significantly increased the relative abundances of *Mortierella* and *Linnemannia* by 68.45 and 139.44%, respectively, whereas the relative abundances of *Tausonia* and *Pseudogymnoascus* were significantly decreased by 42.12 and 69.31%, respectively, compared to the CK treatment. The SBSH treatment significantly increased the relative abundance of *Linnemannia* by 125.01% and decreased the relative abundance of *Metacordyceps* by 36.54% (*p* < 0.05; Figure 3D; Supplementary Table S2). The results of the regression analysis showed that the composition of the bacterial community structure was significantly positively correlated with PMI.1 (*p* < 0.05), while the composition of the fungal community structure had significant negative correlations with AMI and PMI.1 (*p* < 0.05) (Supplementary Figure S3).

**Figure 4:**
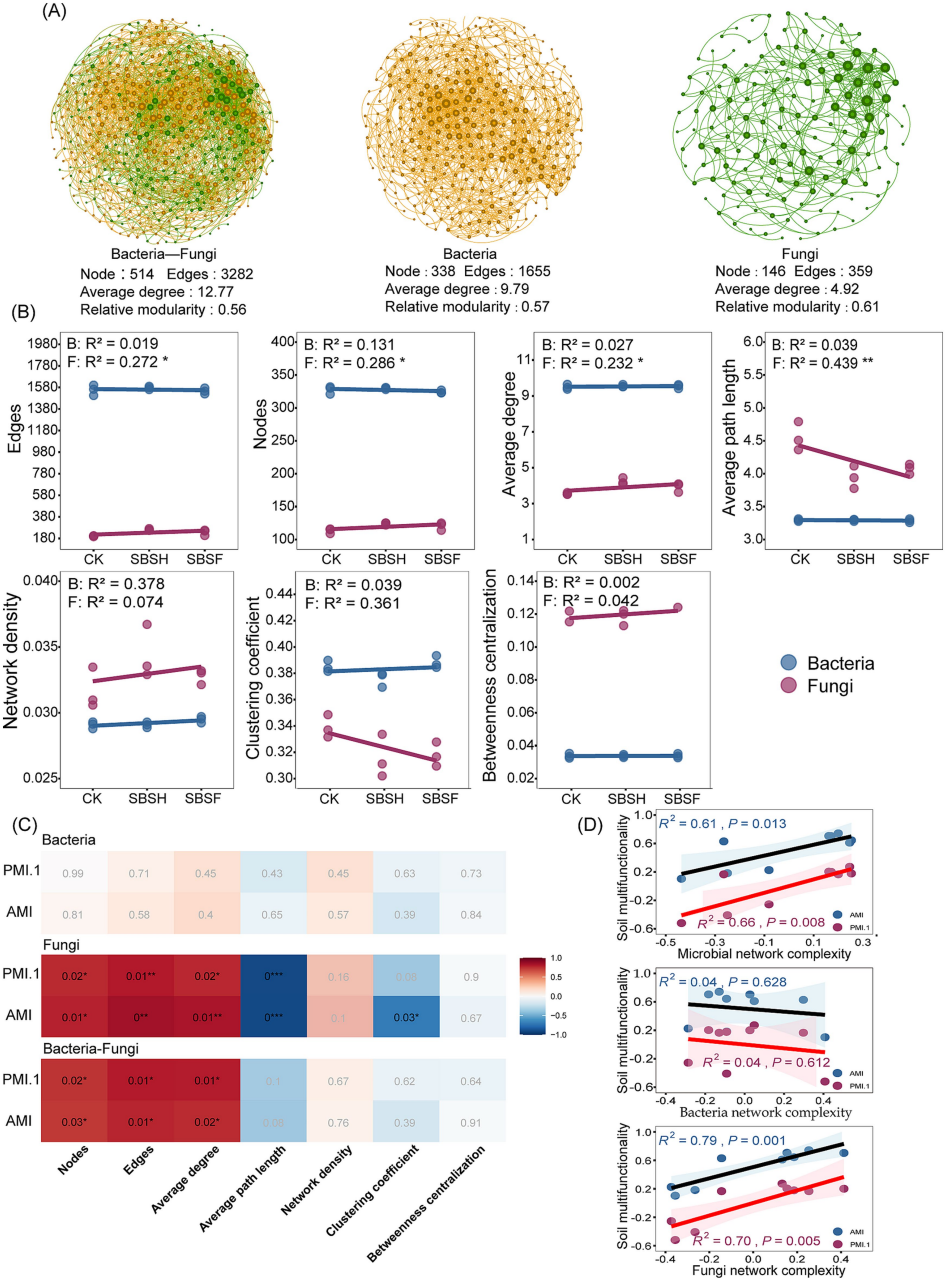
Soil microbial community collinear network **(A)**, microbial co-occurrence network topological properties **(B)**, correlation heat map between soil multifunctionality and subnetwork topological properties **(C)**, and linear regression analysis between soil multifunctionality and microbial network complexity **(D)** under different straw returning amounts. The color in the heat map represents the Spearman correlation; when the color is darker, the correlation is more significant. Significant results are indicated by *p < 0.05, **p < 0.01, ***p < 0.001.

A total of 49 bacterial species and 10 fungal species with significant differences were identified via multilevel species difference discriminant analysis (LEFSe). By setting the threshold of bacteria LDA to ≥2.5 and fungi LDA to ≥2.0 for the difference analysis, we found the rhizosphere soil subjected to the CK treatment was mainly enriched by the genera *Burkholderia*, *Caballeronia*, *Paraburkholderia,* and *Bryobacter*; the SBSH treatment significantly enriched *Segetibacter*, *Hymenobacter,* and *Agrobacterales*; and the SBSF treatment exhibited a wide range of bacterial microbial enrichment, including *o_Rokubacteriales*, *f_Anaerolineaceae*, *Methylotenera*, *RB41*, and *Nitrosospira*. In the fungal community, the CK treatment only enriched *Thelebolus* in the rhizosphere soil; the SBSH treatment enriched *Cyathus*, *Ramicandelaber*, *Leuconeurospora*, and *Scolecobasidium*; and the SBSF treatment significantly enriched *Ophiocordyceps*, *Flagelloscypha*, *Gigaspora*, *Degelia*, and *Lobulomyces* (Supplementary Figure S4).

### 3.4 Properties of the rhizosphere soil microbial networks, and association with multifunctionality

Co-occurrence network analysis revealed a substantial difference in the structures and functions of the bacterial and fungal communities. The microbial network contains 514 nodes and 3,282 relevant edges, the bacterial network contains 338 nodes and 1,655 edges, and the fungal network contains 146 nodes and 359 edges, indicating a substantial number of interactions across domains and that interactions in the bacterial community were denser and more complex than those in the fungal community (Figure 4A).

In order to analyse the variation patterns of the network topology indexes, we extracted the subnetworks of microbial communities under the different treatments (Figure 4B; Supplementary Figure S5). The fungal community network from the SBSH treatment was 9.41, 15.93, and 30.13% greater in terms of nodes, edges, and average degrees, respectively, compared to that of the CK treatment. The average path length from the fungal community network was 15.46% lower (*p* < 0.05). In comparison, the characteristics of bacterial community network topology were not statistically different across the different treatments (*p* > 0.05). Analyses of the characteristics of the microbial community network topologies, conducted on nodes, edges, and positive correlations, indicated that the SBSH treatment increased the number of each by 3.75, 7.08 and 7.46%, respectively, compared to the CK treatment (*p* < 0.05). Relationships between the topology characteristics of the bacterial and fungal subnetworks and soil multifunctionality were evaluated using Spearman correlation analysis (Figure 4C). The results indicated there were no significant relationships between the topological characteristics of bacterial subnetworks and the soil multifunctionality index (*p* > 0.05). The fungal subnetwork’s topological properties for nodes, edges, and average degree were significantly positively correlated with both the soil AMI and the principal coordinate multifunctionality index (*p* < 0.05), whereas average path length was significantly negatively correlated with the soil AMI and the principal coordinate multifunctionality index (*p* < 0.05). Furthermore, the nodes, edges, and average degree of the microbial community’s subnetwork topology characteristics were significantly positively correlated with both the soil AMI and the principal coordinate multifunctionality index (*p* < 0.05). Regression analyses indicated a significant positive relationship between the complexity of fungal and microbial networks and the soil multifunctionality index (*p* < 0.05; Figure 4D), in which the complexity of the fungal network was significantly positively correlated (*p* < 0.01) but the complexity of the bacterial network was not significantly correlated with the soil multifunctionality index (*p* > 0.05).

### 3.5 Rhizosphere soil microorganisms drive soil multifunctionality changes and productivity improvements

**Figure 5:**
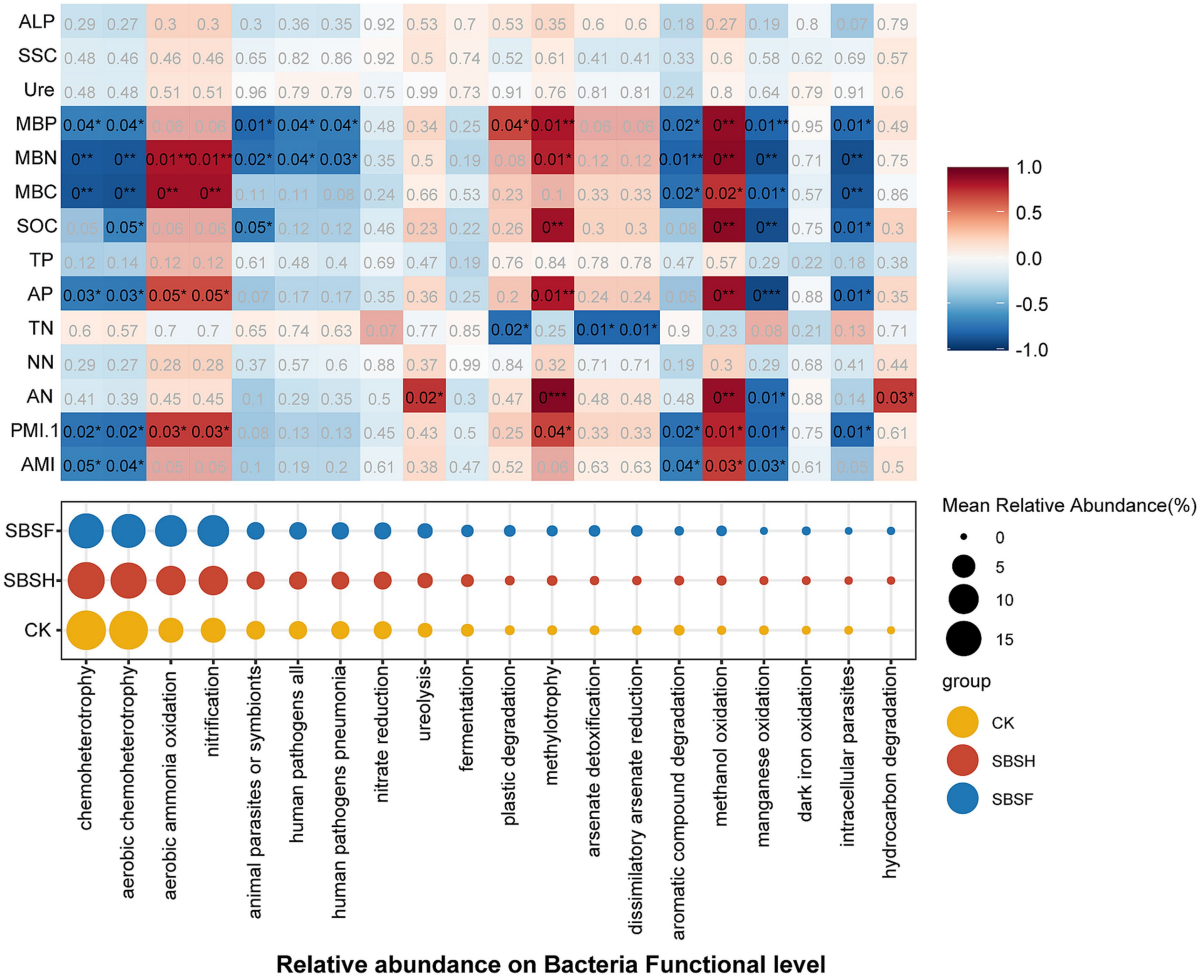
Prediction of soil bacterial function and its correlation with soil multifunctionality. The size of the circle represents the average relative abundance (%) of bacterial function. Significant results are indicated by *p < 0.05, **p < 0.01, ***p < 0.001.

The FAPROTAX tool was utilized to analyse the functions of the bacterial community because robust ASV classification results of were obtained, enabling further investigation of the relationship between microbial functional characteristics and soil multifunctionality. The results from the functional prediction analysis were annotated to 45 biological functions, and of those functions, 12 functions had statistically significant differences (*p* < 0.05). Further analysis of the biological functions of the top 20 relative abundances indicated that the SBSF treatment significantly reduced the functional abundance of chemoheterotrophy and aerobic chemoheterotrophy and significantly increased the functional abundance of methylotrophy and methanol oxidation (*p* < 0.05) compared with the CK treatment. Correlation analysis suggested that soil microbial biomass (MBC, MBN, and MBP), SOC, AP, and PMI.1 exhibited stronger correlations with bacterial biological functions, even though the abundance of chemoheterotrophy and aerobic chemoheterotrophy were significantly negatively correlated with the functional index (*p* < 0.05). In contrast, some specific functions, including aerobic ammonia oxidation, nitrification, methylotrophy, and methanol oxidation, were significantly positively correlated with the soil functional index (*p* < 0.05; Figure 5). Additionally, the current study performed a fungal function prediction analysis with the FUN Guild online data platform (Supplementary Figure S6). The biological functions of the top 20 fungi were predominantly saprophytic fungi, followed by pathogenic fungi and symbiotic fungi. The relative abundances of animal pathogens, animal pathogen–soil saprotroph systems, and animal pathogen–plant pathogen–undefined saprotroph systems in the CK treatment were higher than those in the straw-returning treatments (*p* < 0.05). By contrast, the relative abundances of dung saprotroph–plant saprotroph and animal pathogen–plant pathogen–undefined saprotroph systems in the SBSH treatment were significantly higher than those in the CK treatment, whereas the relative abundance of *Arbuscular mycorrhiza* in the SBSF treatment significantly increased (*p* < 0.05). The correlation analysis further shows that plant pathogens, animal pathogen–plant pathogen–undefined saprotroph systems, animal pathogen–endophyte–plant pathogen–wood saprotroph systems, dung saprotroph–plant saprotroph systems, and *Arbuscular mycorrhiza* were significantly positively correlated with the soil functional index (*p* < 0.05). Moreover, animal pathogens, animal pathogen–soil saprotroph systems, dung saprotroph–endophyte–undefined saprotroph systems, and ectomycorrhizal relationships were significantly negatively correlated with the soil functional index (*p* < 0.05).

**Figure 6:**
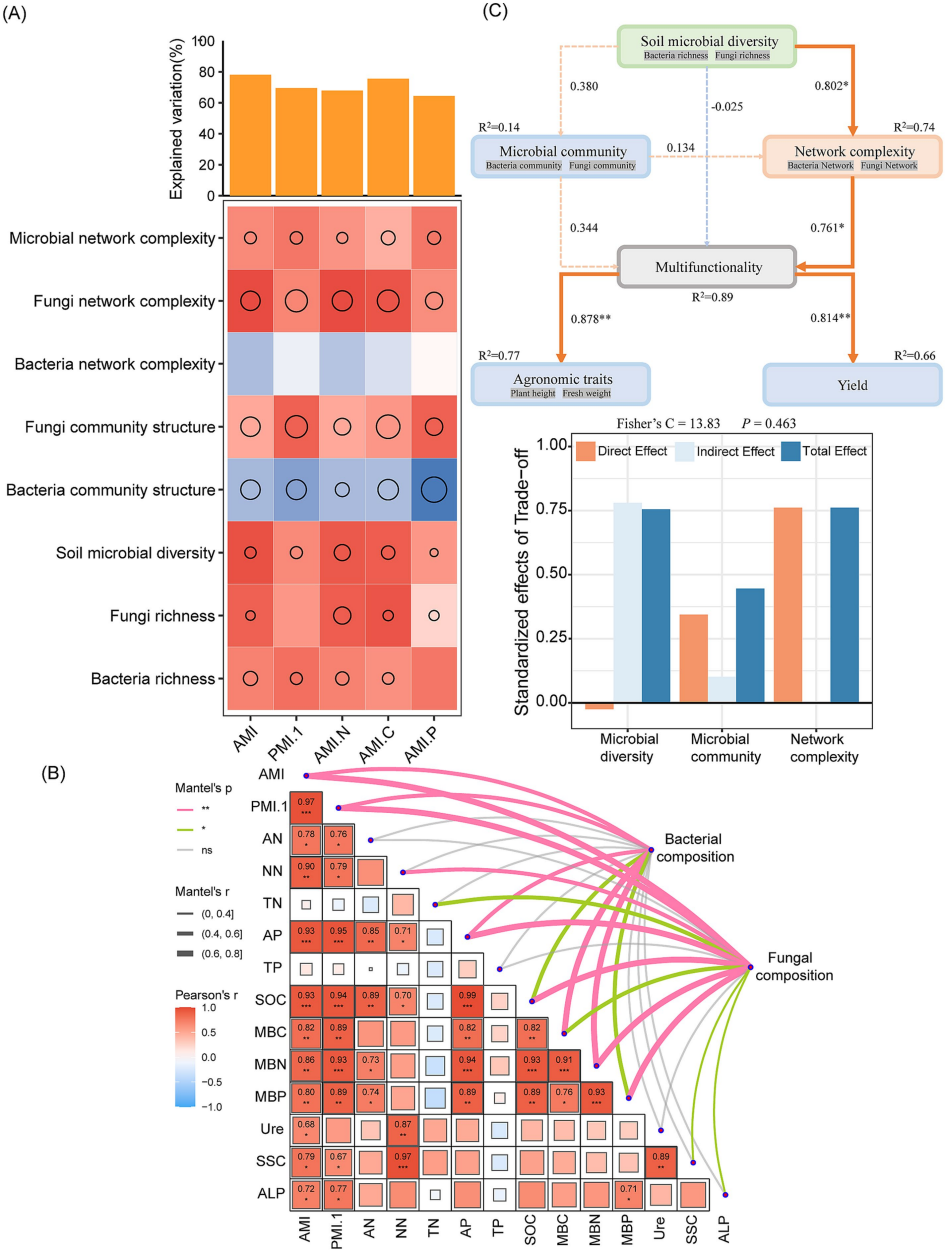
Rhizosphere soil microorganisms drive soil multifunctional changes and productivity improvement. **(A)** The contribution of multiple factors to soil multifunctionality based on Spearman correlation and RF model. The size of the circle represents the percentage increase in the mean square error calculated by the RF model, and the color represents the Spearman correlation. When the color is darker, the correlation is more significant. AMI is the Average Multifunctionality Index, PMI.1 is the Principal Coordinate of Multifunctionality Index, AMI. N is the Nitrogen Cycling Multifunctionality Index, AMI. C is the Carbon Cycling Multifunctionality Index, and AMI. P is the Phosphorus Cycling Multifunctionality Index. **(B)** Mantel analysis of the relationship between microbial composition and soil multifunctional. **(C)** The red solid line arrow represents positive correlation, the blue solid line represents negative correlation, the dotted line represents no correlation, the value on the arrow represents the standard path coefficient, the thickness of the arrow represents the correlation, and R^2^ represents the path interpretation. Significant results are indicated by *p < 0.05, **p < 0.01, ***p < 0.001.

RF analysis (Figure 6A) showed that microbial factors explained 78.08 and 75.54% of the average multifunctionality index (AMI) and C multifunctionality (AMI. C), respectively, values higher than those pertaining to the other functional indices. Bacterial network complexity and community structure were negatively correlated with soil functional index, whereas bacterial community structure was significantly negatively correlated with P multifunctionality (AMI. P) (*p* < 0.01). Rhizosphere soil microbial network complexity, fungal network complexity, fungal community structure, bacterial community structure, and soil microbial diversity are the key biological factors driving soil multifunctional changes. Among these factors, microbial network complexity, fungal network complexity, and bacterial community structure had the most significant effects on soil multifunctionality. Mantel analysis (Figure 6B) results showed that bacterial composition was significantly positively correlated with AMI, PMI.1, AP, MBC, and MBN (*p* < 0.01), and significantly correlated with SOC and MBP (*p* < 0.05). Fungal composition was highly significantly correlated with AMI, PMI.1, NN, AP, SOC, MBN, and MBP (*p* < 0.01), and it also showed significant correlations with TN, MBC, SSC, and ALP (*p* < 0.05). Compared with no straw return, both half and full amounts of soybean straw return significantly increased plant height, fresh weight, and yield (*p* < 0.05). However, no significant differences were observed in these parameters between the half and full amounts of return (*p* > 0.05; Supplementary Figure S7). The SEM (Figure 6C) showed that network complexity was a significant positive direct factor driving the change in soil multifunctionality (std estimate = 0.761, *p* < 0.05); soil microbial diversity had significant positive indirect effects on soil multifunctionality by regulating network complexity (std estimate = 0.802, *p* < 0.05). In addition, soil multifunctionality exhibited significantly positive regulatory effects on crop growth (std estimate = 0.878, *p* < 0.01) and yield (std estimate = 0.814, *p* < 0.01).

## 4 Discussion

### 4.1 Straw returning substantially increased soil microbial diversity and soil multifunctionality

**Figure 7:**
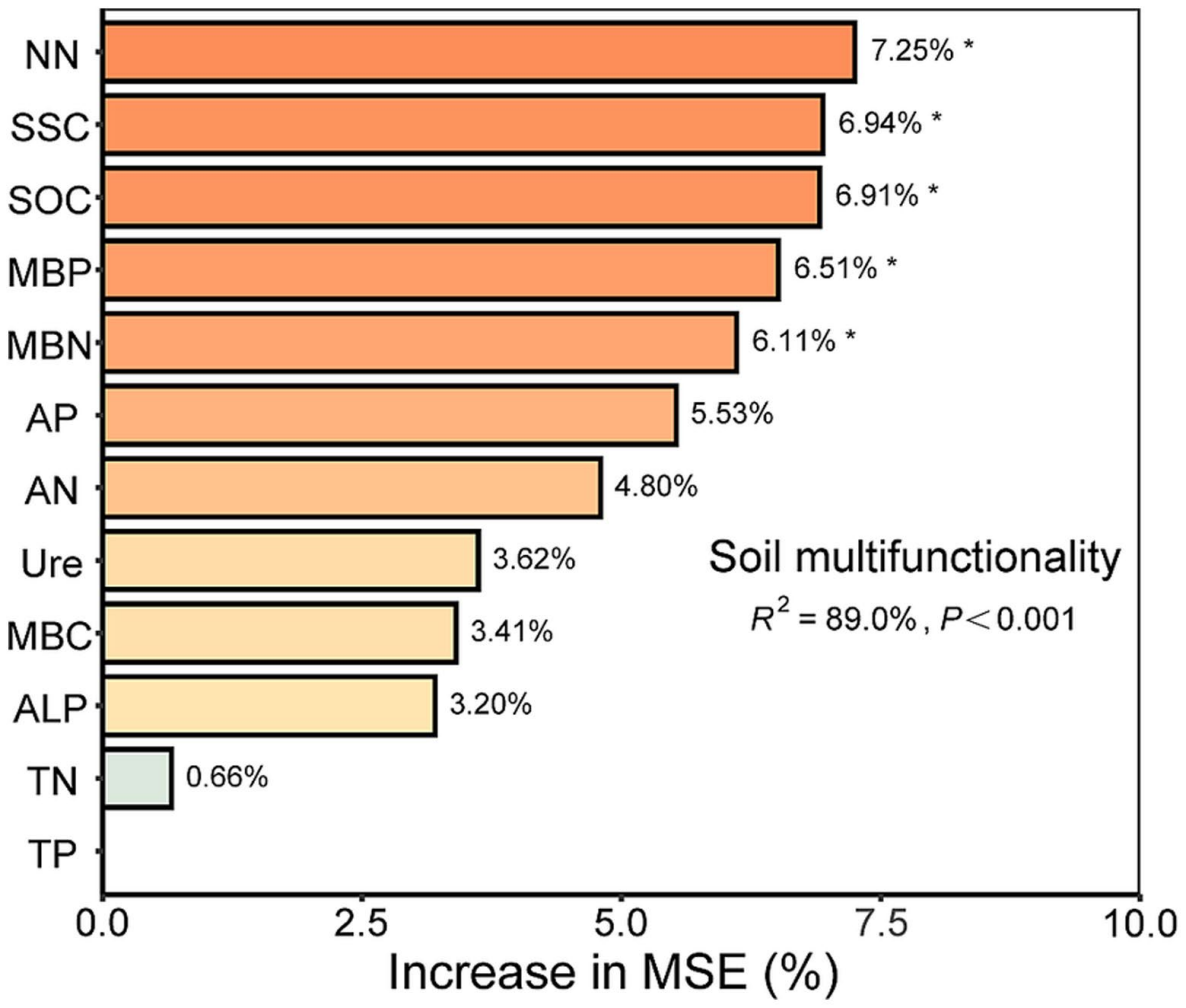
Predicting the importance of environmental factors to soil average multifunctionality based. ALP, Alkaline phosphatase; AP, Available phosphorus; TP, Total phosphorus; MBP, Soil microbial biomass Phosphorus; AN, Ammonium nitrogen; NN, Nitrate nitrogen; MBN, Soil microbial biomass nitrogen; TN, Total nitrogen; Ure, Urease; MBC, Soil microbial biomass carbon; SOC, Organic carbon; SSC, Soil sucrase. Significant results are indicated by *p < 0.05.

Returning soybean straw led to a significant increase in soil microbial diversity and multifunctionality, and the impact of soil bacteria and fungi as a whole (microbial diversity) on multifunctionality was greater. Studies have shown that there is a significant positive relationship between soil microbial diversity and soil multifunctionality. Improvements in soil microbial diversity can enhance nutrient availability, improve microbial growth efficiency, and enhance organic matter decomposition (Delgado-Baquerizo et al., 2016b; Wagg et al., 2014; Wang X. et al., 2023). Soybean straw returning provides abundant organic substrates for soil microorganisms and effectively alleviates soil nutrient limitations (Zhao et al., 2016). Consequently, metabolic intermediates and final products after straw decomposition further provide nutrients for microorganisms. In addition, straw’s physical structure helps regulate soil pores, improve soil aeration and water conditions, and create a good environment for the activity, growth, and reproduction of microbial life. These conditions jointly improve soil microbial diversity, thereby enhancing soil nutrient cycling, organic matter decomposition, and structural stability, ultimately enhancing soil versatility (Li et al., 2022). Moreover, the results of RF model analysis verified this conclusion (Figure 7). Soil SSC and SOC content were important predictors of soil multifunctionality (*p* < 0.05). Straw-returning treatment significantly increased the abundance of functional genes related to nitrogen fixation, nitrification, and denitrification and effectively promoted the transformation and utilization efficiency of nitrogen, which is consistent with the positive effects of nitrate nitrogen and microbial biomass nitrogen on soil multifunctionality (*p* < 0.05) (Ding et al., 2018). The effects of MBP on soil multifunctionality may be related to soybean planting. Meanwhile, the phosphorus uptake of soybeans is 1.3–1.7 times large than that of corn and wheat (Wang et al., 2022). Additionally, under straw-returning treatment, the number of functional genes associated with phosphorus solubilization was significantly higher, which further enhanced the release of soil-fixed phosphorus and met the phosphorus needs of the crop (Ding et al., 2018). This treatment can thus influence soil function profoundly and is an important factor influencing soil multifunctionality. Although both half (SBSH) and full (SBSF) straw return significantly increased the soil multifunctionality index, the half-return treatment better maintained microbial community stability (Figure 1A). This indicates that moderate straw input may sustain community stability by avoiding intense microbial competition induced by excessive resource input. The differential stability responses to straw return rates can be further interpreted through the distinct functional roles and response strategies of bacteria and fungi during decomposition. As a strong environmental disturbance, the amount of straw input directly shapes resource structure and niche partitioning, thereby differentially influencing bacterial and fungal communities with their contrasting life-history strategies. The full straw return (SBSF) introduces a large quantity of labile organic matter in a single event, which may lead to rapid bacterial growth and intense resource competition. Such pronounced community succession is unfavorable for maintaining microbial homeostasis (Li et al., 2024). In contrast, the half straw return (SBSH) provides a moderate level of resource input that enhances microbial activity without causing competitive imbalance due to resource surplus, thereby supporting higher community stability (Liu et al., 2022). The significantly increased complexity of the fungal network under the SBSH treatment (Figure 4B) further supports this interpretation. Moderate disturbance creates favorable conditions for the development of fungal networks, which possess a stronger capacity to maintain homeostasis, whereas full straw return may primarily stimulate the bloom of certain fast-growing bacterial taxa (Xu et al., 2023). Simultaneously, differences in the soil micro-environment induced by the rate of straw returning also significantly influence microbial community dynamics. Straw returning, through its physical covering effect, effectively buffers soil temperature fluctuations and conserves moisture, with the application rate directly determining the intensity of this micro-environmental regulation (Wang et al., 2025). Full straw return may lead to excessively high soil moisture and reduced aeration in the short term. Such a pronounced shift in the micro-environment might preferentially favor the proliferation of certain moisture-tolerant, anaerobic, and fast-growing bacteria, thereby intensifying resource competition and triggering community fluctuations (Xu et al., 2024). In contrast, half straw return provides moderate coverage, potentially achieving a better balance between improving the micro-environment and maintaining soil aeration. This offers more stable habitat conditions for a wider range of microbial taxa, including fungi (Wang et al., 2025).

### 4.2 Straw returning regulates soil function by regulating microbial community composition and functional microbial enrichment

The soil microbial community plays an irreplaceable role in driving nutrient cycling, maintaining soil structural stability, and regulating soil ecological functions. The community composition and diversity characteristics of soil are highly sensitive to environmental changes, which can accurately reflect subtle changes in soil quality, and are important biological indicators for evaluating soil ecosystem functions (Dong et al., 2017). This study found that soybean straw returning significantly increased the relative abundance of bacterial microorganisms relevant to the carbon and nitrogen cycles in soil. Norank_f_Nitrososphaeraceae is an important class of ammonia-oxidizing archaea, which plays an important role in the nitrogen cycle—they promote the nitrogen cycle by oxidizing ammonia (NH_3_) to nitrite (NO₂^−^) (Tourna et al., 2011). This nitrite can serve as a precursor for nitric oxide (NO), a vital signaling molecule in plants that regulates growth and stress responses (Sun et al., 2015). *Norank_c_MB-A2-108* is a bacterial taxonomic unit that is not clearly classified at the genus level but belongs to the MB-A2-108 class. Some studies have shown that members of the MB-A2-108 class may play a role in organic carbon degradation (Anantharaman et al., 2016). *Rubrobacter* is a bacterium belonging to Actinobacteria that plays a role in biomass degradation and the carbon cycle (Carreto et al., 1996). Soil fungi play an important role in nutrient cycling, organic matter degradation, and biological control (Lu et al., 2025). Tang et al. (2023) found that the composition of the fungal community was mainly affected by SOC. Returning soybean straw, as exogenous organic matter, enhanced the deterministic process of soil fungal community construction; that is, fungi that easily degrade straw were enriched in large quantities. They occupy a high niche and exhibit obvious growth advantages.

LEFSe analysis indicated significant differences in the bacterial and fungal communities enriched in rhizosphere soil under different straw-returning treatments. Taxa such as *Burkholderia*, *Caballeronia*-*Parburkholderia*, and *Bryobacter*—belonging to Proteobacteria and Acidobacteria—and *Thelebolus* fungi were mainly enriched in the rhizosphere soil subjected to the CK treatment. These microorganisms play a role in organic matter decomposition, nutrient cycling, and symbiotic nitrogen fixation (Devi et al., 2020; Lian et al., 2025). In contrast, the SBSH treatment significantly enriched bacteria such as *Segetibacter* and *Hymenobacter*, and fungi such as *Cyathus* and *Ramicandelaber*—of the phyla Bacteroidetes, Basidiomycota, and Zygomycota-which not only degrade organic matter but also possibly have a greater association with the promotion of plant growth and stress resistance potential (Puget et al., 1998; Sathya et al., 2017; Tang et al., 2015). The SBSF treatment exhibited more diverse microbial community characteristics, having enriched bacteria such as o_Rokubacteriales, f_Anaerolineaceae, *Methylotenera*, and *Nitrosospira*, as well as fungi such as *Ophiocordyceps* and *Gigaspora*, which belong to Armatimonadetes, Chloroflexi, Proteobacteria, Nitrospirae, Ascomycota, and Glomeromycota. Their functions are more diversified and include nitrogen cycling, methane oxidation, pathogen inhibition, and soil structure improvement (Xi et al., 2022; Yao et al., 2025).

### 4.3 Straw returning drives soil multifunctionality by enhancing fungal network complexity

Microbial communities and their interactions play a role critical to both the formation and maintenance of the functions and services of soil ecosystems. A mixture of microbial species interacting with each other synergistically drives the functions of the soil ecosystem. Microbial groups and their network structures positively influence soil ecosystems, which contributes to soil multifunctionality (Zhang J. et al., 2022). In this case, co-occurrence network analysis indicated distinct structural and ecological functional differences between bacterial and fungal communities. The overall microbial network identified complex cross-domain interaction features, in which the bacterial community had more nodes and edges than the fungal community, indicating that interactions within the bacterial community were more intense. Studies have shown that bacterial communities often form dense networks through competitive or synergistic metabolic strategies, whereas fungal communities exhibit relatively low network complexity, owing to niche differentiation and functional specificity (Bahram et al., 2021; Zhan et al., 2021). Following straw return, microbial assemblages became more compact and the network environment more complex, with different straw return rates exerting differential regulation on this interactive environment. Although bacterial communities maintained higher network density across all treatments, their topological characteristics showed no significant differences among different return rates. In contrast, the half straw return (SBSH) treatment most significantly enhanced the fungal network, increasing the number of nodes, edges, and average degree by 9.41, 15.93, and 30.13%, respectively. When soil fertility and quality improved due to straw return, metabolic activities of various microorganisms intensified, enabling them to occupy more ecological niches after environmental filtering. This explains why closely related microbial species can coexist—inefficient microorganisms are replaced by more efficient taxa (Yu et al., 2019). This may account for the most significant enhancement of fungal network topological properties under the SBSH treatment with moderate resource input. Some studies suggest that excessive straw return may trigger intense competition for microbial resources, while moderate return creates optimal conditions for community assembly (Li et al., 2024; Liu et al., 2022). Furthermore, studies have confirmed that the enhancement of fungal networks may directly improve soil multifunctionality by promoting soil aggregate formation and nutrient cycling through hyphal extension (Ma et al., 2024). Regression analysis in this study also confirmed that only the complexity of the fungal network showed a significant positive correlation with the soil multifunctionality index (*p* < 0.01), while no such relationship was observed for the bacterial network. Therefore, we propose that soybean straw return, particularly half return, primarily enhances soil multifunctionality by specifically increasing the complexity of the fungal network in the rhizosphere soil. Complex soil microbial networks facilitate the maintenance of ecosystem functions, as diverse network interactions provide advantages in resource utilization and information transfer for multiple ecological functions (Ma et al., 2024). If soil microbial community composition becomes simplified and complexity decreases, ecosystem functions will be impaired, and this impact will intensify over time (Xiao K. et al., 2023). In summary, the advantage of half straw return lies in creating a moderate resource environment that optimally drives a functional enhancement pathway centered on fungal network complexity, thereby achieving synergistic improvements in soil multifunctionality and microbial community stability.

### 4.4 Mechanism driving rhizosphere soil multifunctionality changes

Herein, the relationship between microbial biological function and soil multifunctionality under different straw-returning treatments was explored via the predictive analysis of bacterial and fungal functions. The SBSF treatment significantly decreased the functional abundance of chemoheterotrophy and aerobic chemoheterotrophy and significantly increased the functional abundance of methylotrophy and methanol oxidation, suggesting that straw return may change how sources of carbon are utilized. Cheomoheterotrophy and aerobic chemoheterotrophy are typical heterotrophic metabolic functions that depend on the decomposition and utilization of organic carbon. Straw returning may change the composition and availability of SOC, decreasing the abundance of heterotrophic functions that depend on complex organic carbon. These results are consistent with the research results of Wu et al. (2021). By contrast, the increase in methylotrophy and methanol oxidation may be related to the utilization of simple carbon sources such as methanol produced during straw degradation (Shu et al., 2022). The outcomes of the functional analysis of fungal communities revealed that the SBSH treatment significantly increased the abundance of dung saprotroph–plant saprotroph systems and the SBSF treatment significantly increased the abundance of *Arbuscular mycorrhiza*, indicating that straw-returning treatments had a significant influence on the biological functions of fungi, particularly through increasing the proportions of symbiotic and saprophytic bacteria. Saprophytic fungi have the ability to decompose substantial amounts of refractory carbon and are closely associated with the carbon cycle (Deng et al., 2019). The increase in *Arbuscular mycorrhiza* may be related to improved soil phosphorus availability because these types of fungi can promote the absorption of phosphorus by expanding the range of root absorption (Nie et al., 2018). In contrast to the straw-returning treatments, the CK treatment significantly enriched animal pathogens, animal pathogen–soil saprotroph systems, and animal pathogen–plant pathogen–undefined saprotroph systems. These enriched pathogens showed a significant negative correlation with microbial richness and fungal network complexity (*p* < 0.05; Supplementary Figure S8), indicating that when they occupy dominant ecological niches, they can suppress soil microbial diversity, disrupt the stability of microbial communities, and reduce their resistance to disturbance (Nishisaka et al., 2025). This inhibitory effect is particularly pronounced in monoculture systems due to the weakened “pathogen dilution effect” resulting from low biodiversity (Wang G. et al., 2023). In summary, straw returning introduces organic carbon, promotes the development of beneficial fungal and bacterial functional groups, and facilitates the formation of structurally complex and functionally diverse microbial communities. This effectively suppresses the enrichment of pathogens and their negative impacts through ecological niche competition, to alleviate the pathogen enrichment caused by continuous soybean monoculture.

RF analysis indicated that rhizosphere soil microorganisms exerted a significant impact on the soil AMI and the C functional index. Also, the bacterial community structure was significant and negatively correlated with the P functional index. The complexity of rhizosphere soil microbial communities and networks, fungal community structure, bacterial community structure, and soil microbial diversity were biological factors that most influenced changes in the soil multifunctionality index. Ultimately, the complexity of these networks and community structures had significant effects on soil multifunctionality. As essential components of the soil microbial community, soil fungi and bacteria, and their interactions impact our understanding of soil multifunctionality; however, more work is needed before we will be able to anticipate what soil fungi and bacteria will do collectively and separately. To further elaborate on the mechanisms by which rhizosphere soil fungal and bacterial communities collectively influence soil multifunctionality, we utilized SEM to further examine how the diversity, community structure composition, and network complexity of bacteria and fungi affect soil multifunctionality while also looking into the influence of soil multifunctionality on product improvement. Network complexity was significant as a positive direct factor impacting the change in soil multifunctionality (std estimate = 0.761). The effects of soil microbial diversity on soil multifunctionality did not result from significant direct effects; rather, they exhibited significant positive indirect effects on soil multifunctionality by influencing network complexity (std estimate = 0.802). The influence of microbial network complexity on multifunctionality was more significant than the influence of microbial community structure and indicates that microbial complexity better predicts and positively affects soil multifunctionality than microbial diversity and community structure. The effects of fungal complexity were greater than the effects of bacterial complexity. It has been hypothesized that the complexity of soil microbial co-occurrence networks affects soil variability more significantly than microbial diversity, composition, and community structure (Ma et al., 2024).

The results of this study elucidate the mechanism through which soybean straw returning in black-soil regions enhances soil multifunctionality and crop productively—by regulating the complexity of the rhizosphere microbial network. However, as these conclusions are derived from a specific crop–straw–soil system, further validation across diverse agricultural systems is required to make them generally applicable. The biological nitrogen fixation capacity unique to leguminous crops may significantly alter carbon and nitrogen cycling processes and microbial interaction patterns in the rhizosphere microdomain, representing a fundamental characteristic of the rhizosphere environment of non-leguminous crops. Root-secreted signaling compounds, such as flavonoids, in leguminous plants may specifically recruit the microbial taxa involved in nitrogen fixation or organic nitrogen activation, thereby fostering a distinct interaction network (Zhang W. et al., 2022). In contrast, in systems dominated by gramineous crops, microbial communities may exhibit stronger responses to straw with higher carbon-to-nitrogen ratios (e.g., wheat or rice straw). The decomposition process of such residues could induce more intense competition for nitrogen between microorganisms and crops, consequently influencing microbial network structure and functional expression (Shi et al., 2024). Therefore, the “straw returning–microbial network modulation–productivity enhancement” model proposed in this study necessitates further validation across a broader range of crop types, straw varieties, and soil conditions. Such efforts are critical to clarifying the universal mechanisms by which microbial networks drive soil multifunctionality and to providing a theoretical basis for the formulation of tailored straw-returning strategies for different agricultural systems.

## 5 Conclusion

The results of this study reveal the microbiological mechanisms underpinning the impact returning soybean straw to the field in black-soil areas has on improving soil versatility by reshaping the microbial community and network structure of the crop’s rhizosphere soil. The results indicate that soybean straw returning significantly increased soil microbial diversity and versatility, and there were significant differences in the microbial groups enriched in the rhizosphere under different straw-returning treatments. Co-occurrence network analysis indicated that soybean straw returning increased soil multifunctionality by enhancing the complexity of rhizosphere soil microbial networks, and this increase in complexity was mainly driven by fungal networks. Microbial network complexity is a direct and key factor in improving soil multifunctionality, whereas microbial diversity indirectly enhances soil multifunctionality by regulating microbial network complexity. Moreover, SEM analysis showed that the improvement in soil multifunctionality driven by microorganisms played an important role in improving crop yield. Our comprehensive results demonstrate that under soybean straw return conditions, half-dose straw returning represents the optimal approach for enhancing soil microbial community stability, improving ecosystem multifunctionality, and achieving sustainable high crop yields. This study provides a theoretical basis for the development of a “straw returning–microbial network regulation–soil function improvement–crop yield increase” agricultural management model and has important scientific significance and practical value for soil health regulation and straw resource utilization in black-soil areas.

## Data Availability

The datasets presented in this study can be found in online repositories. The names of the repository/repositories and accession number(s) can be found in the article/Supplementary material.
