# Supplementary material for: How straw returning impacts microbial-interaction-network-mediated improvements in soil multifunctionality
Source: Front Microbiol. 2025 Dec 10;16:1710232. doi: 10.3389/fmicb.2025.1710232 (PMC12728582; doi:10.3389/fmicb.2025.1710232)
Supplement: Supplementary file 1 [file Data_Sheet_1.docx]

Supplementary Material

How Straw Returning Impacts Microbial- Interaction- Net-work-Mediated Improvements in Soil Multifunctionality

**Haogeng Zhao ^1,2^, Zhanyuan Lu ^2^, Meijuan Cheng ^3^, Shuli Wei ^2^, Jing Fang ^3^, Wenqing Cao ^1^, Huimin Shi ^2^, Wei Zhang ^1^, Xiangqian Zhang ^2^, Yan Qu ^2^, Lingyue Liu ^2^, Dejian Zhang ^1^****^,^ * and Xiaoqing Zhao ^2,^ ***

^1^ School of Life Science, Inner Mongolia University, Hohhot 010020, China

^2^Inner Mongolia Academy of Agricultural & Animal Husbandry Sciences/ Key Laboratory of Black Soil Protection and Utilization/ Inner Mongolia Key Laboratory of Degradation Farmland Ecological Restoration and Pollution Control, Hohhot 010031, China

^3^ College of Agronomy, Inner Mongolia Agricultural University, Hohhot 010018, China

^4^ Arong Banner Agricultural Technology Extension Center, Inner Mongolia, Hulun Buir, 162750, China

*** Correspondence:**Xiaoqing Zhao

[zhaoxq204@163.com](mailto:zhaoxq204@163.com)

Dejian Zhang

[111972311@imu.edu.cn](mailto:111972311@imu.edu.cn)

# Supplementary Figures


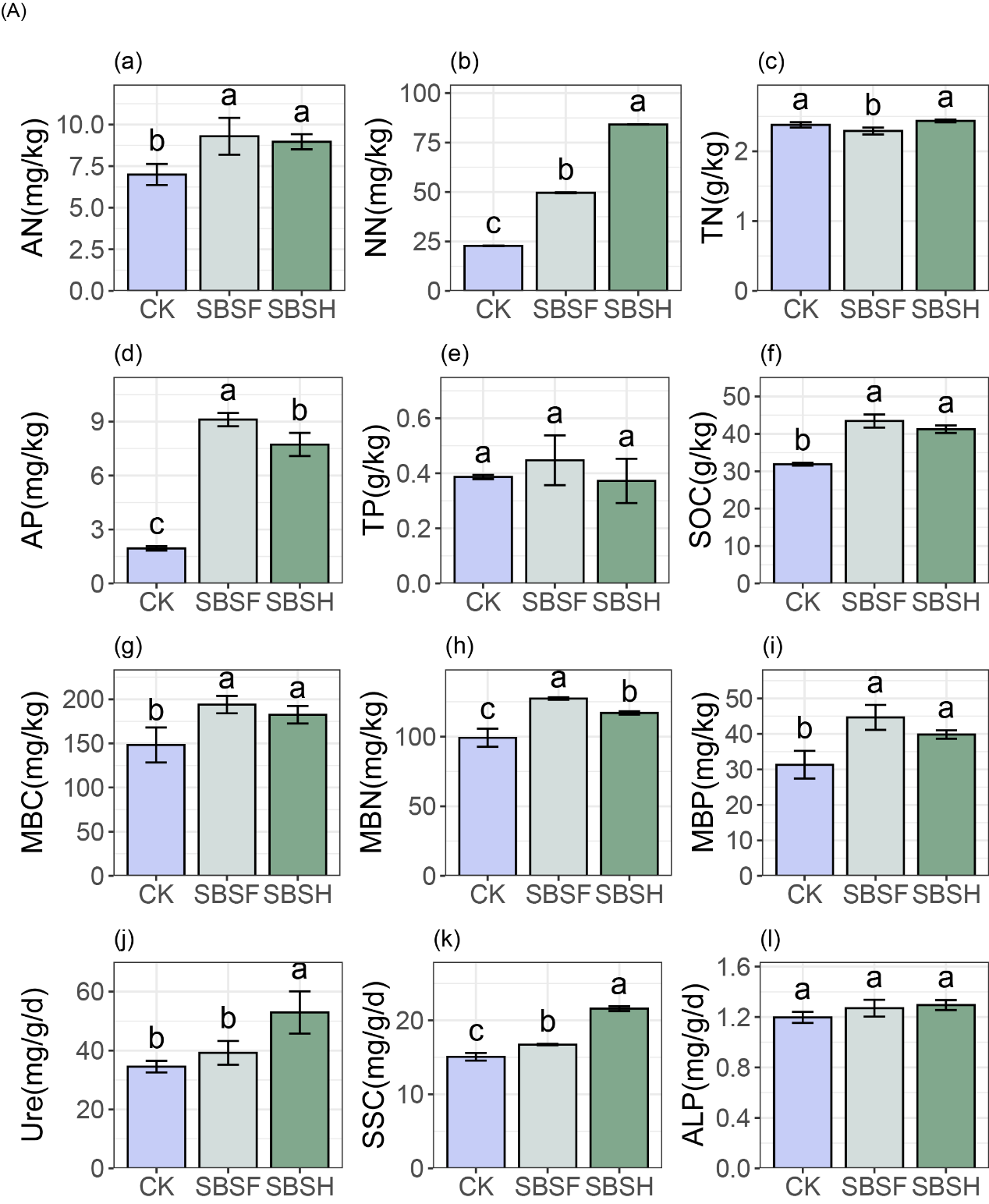


**Supplementary Figure S1.** The variations in soil physicochemical properties under different straw incorporation rates(a～l). AN: ammonium nitrogen; NN: nitrate nitrogen; TN: total nitrogen; AP: available phosphorus; TP: total phosphorus; SOC: organic carbon; MBC: Soil microbial biomass carbon; MBN: Soil microbial biomass nitrogen; MBP: Soil microbial biomass Phosphorus; Ure: urease; SSC: Soil sucrase; ALP: alkaline phosphatase. Different lowercase letters indicate that there are significant differences between different treatments (*p*＜0.05). CK: no-straw returning; SBSH: half-straw returning; SBSF: full-straw returning.


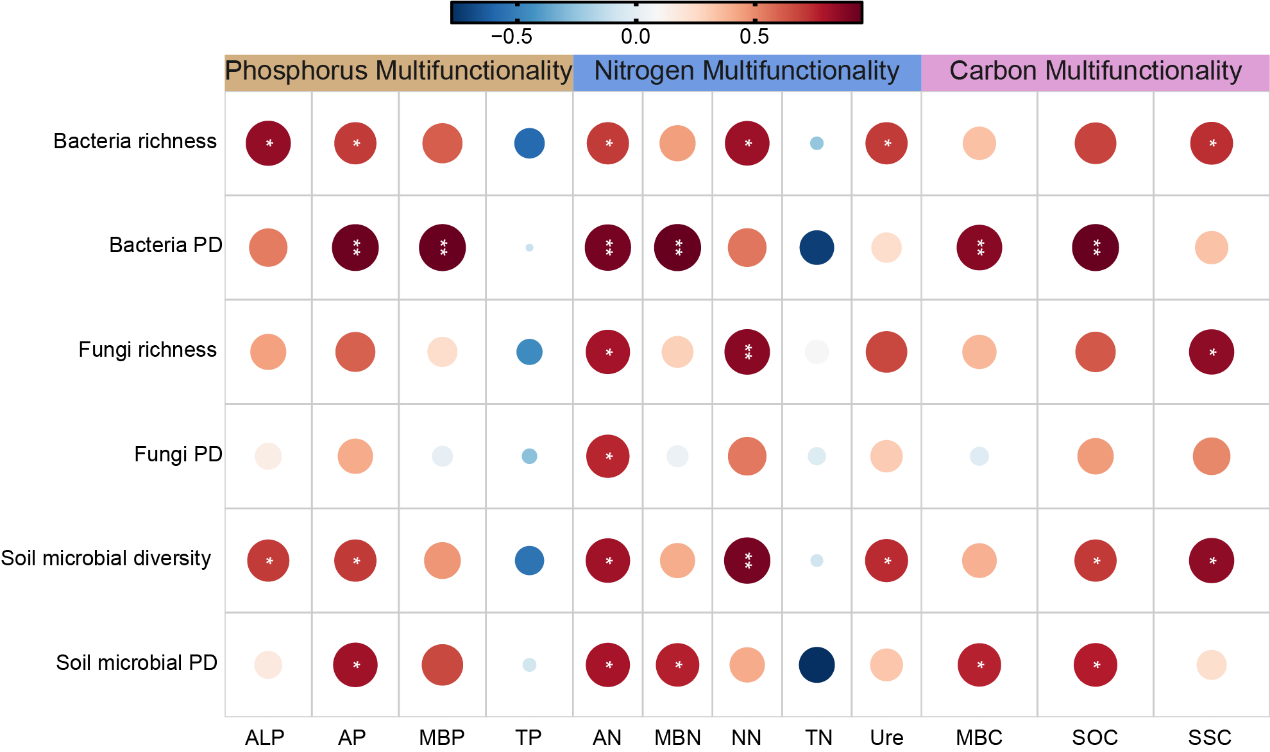


**Supplementary Figure S2.** Correlation between soil microbial diversity and Single function index. AN: ammonium nitrogen; NN: nitrate nitrogen; TN: total nitrogen; AP: available phosphorus; TP: total phosphorus; SOC: organic carbon; MBC: Soil microbial biomass carbon; MBN: Soil microbial biomass nitrogen; MBP: Soil microbial biomass Phosphorus; Ure: urease; SSC: Soil sucrase; ALP: alkaline phosphatase. Blue and red indicate negative and positive correlations between the two variables, respectively. The deeper the color, the stronger the relationship. Significant results are indicated by * *p*<0.05, ** *p*<0.01, *** *p*<0.001. CK: no-straw returning; SBSH: half-straw returning; SBSF: full-straw returning.


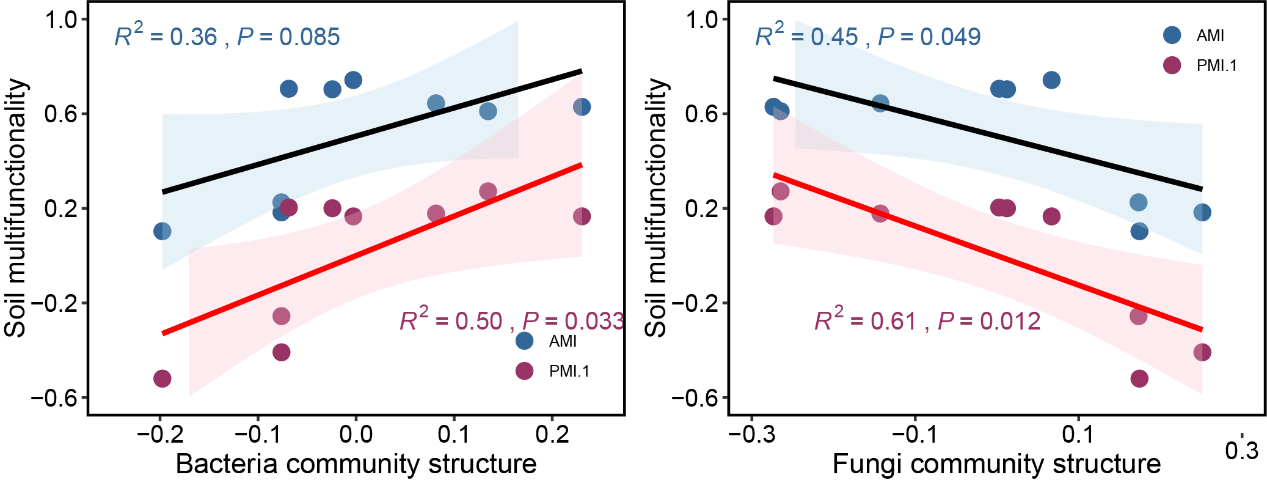


**Supplementary Figure S3.** Regression analysis of the relationships between bacterial/fungal community structures and soil multifunctionality.


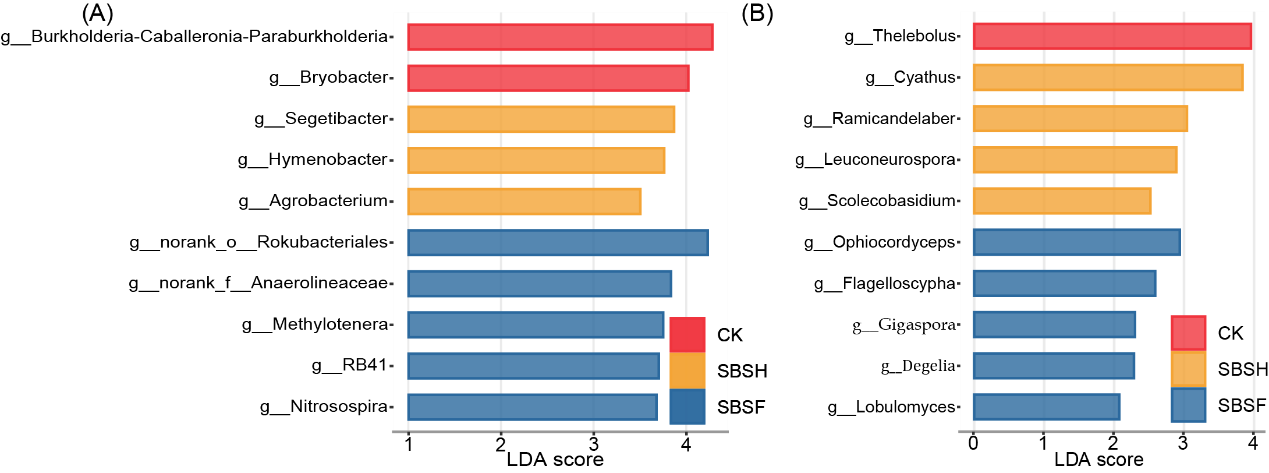


**Supplementary Figure S4.** LEFSe difference analysis of soil microbial community under different straw returning amounts (A, B)


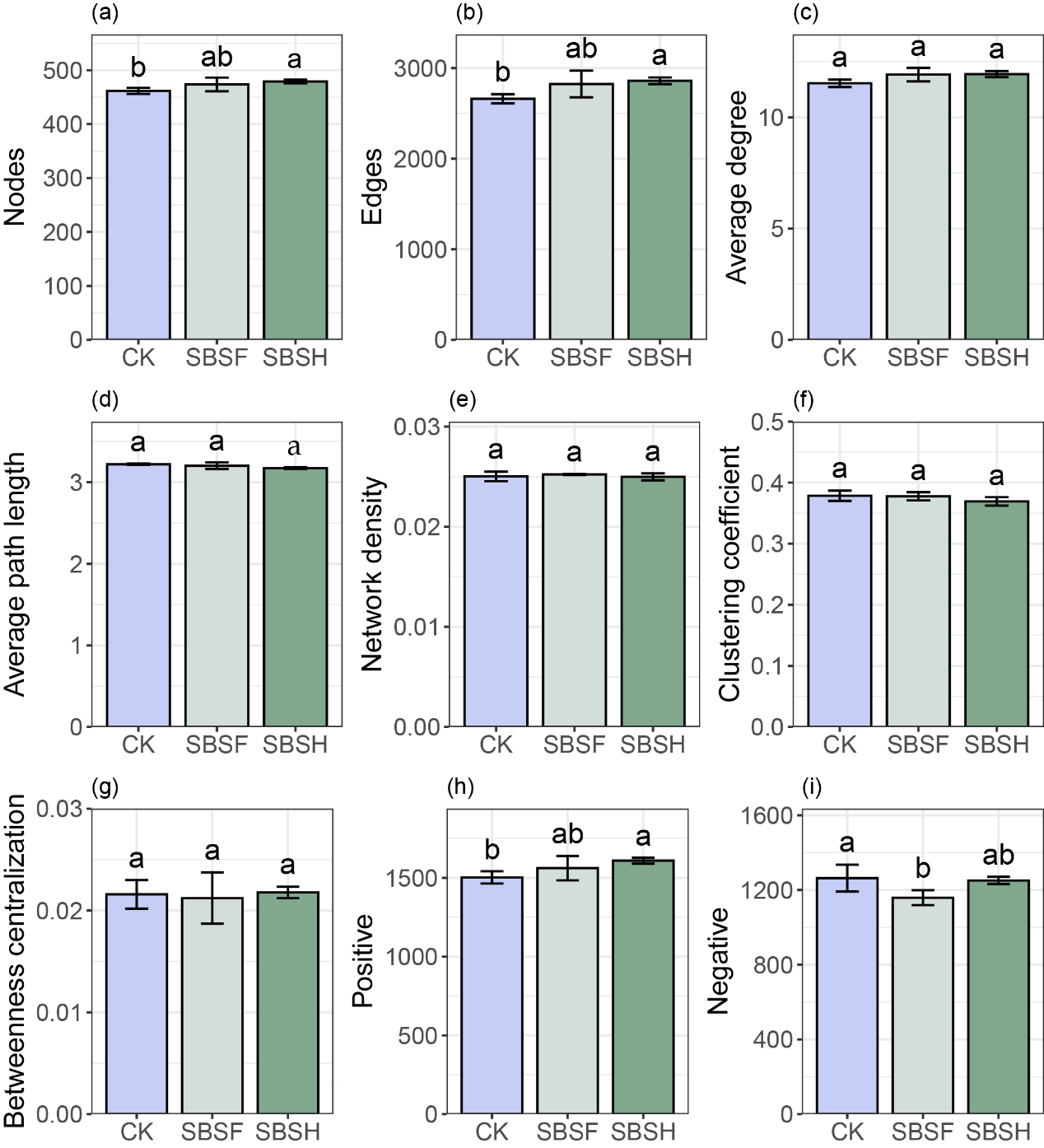


**Supplementary Figure S5.** The topological properties of fungal-bacterial co-occurrence network under different straw returning amounts. Different lowercase letters indicate that there are significant differences between different treatments (*p*＜0.05). CK: no-straw returning; SBSH: half-straw returning; SBSF: full-straw returning.


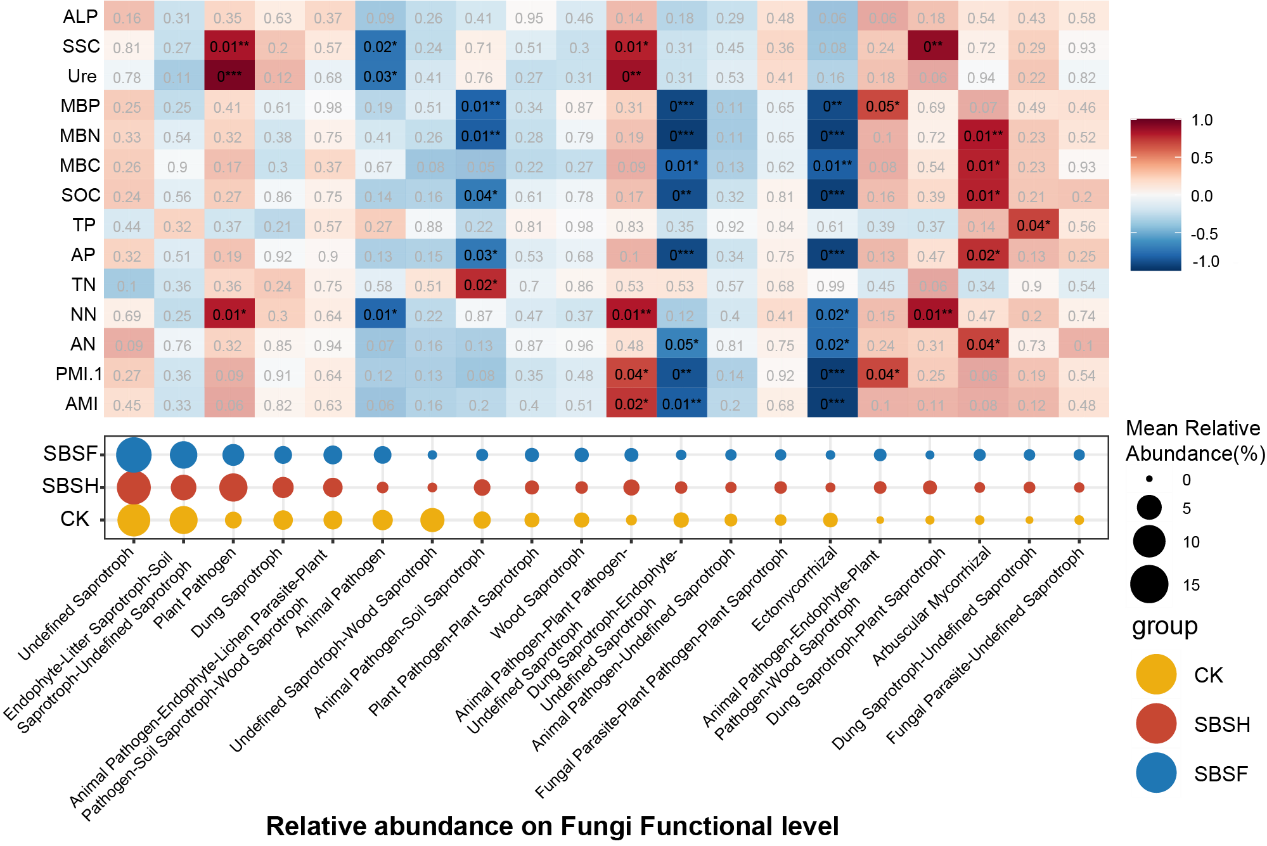


**Supplementary Figure S6.** Prediction of soil fungi function and its correlation with soil multifunctionality. The size of the circle represents the average relative abundance (%) of bacterial function. The color in the heat map represents the Spearman correlation, and the deeper the color, the greater the correlation. CK: no-straw returning; SBSH: half-straw returning; SBSF: full-straw returning.


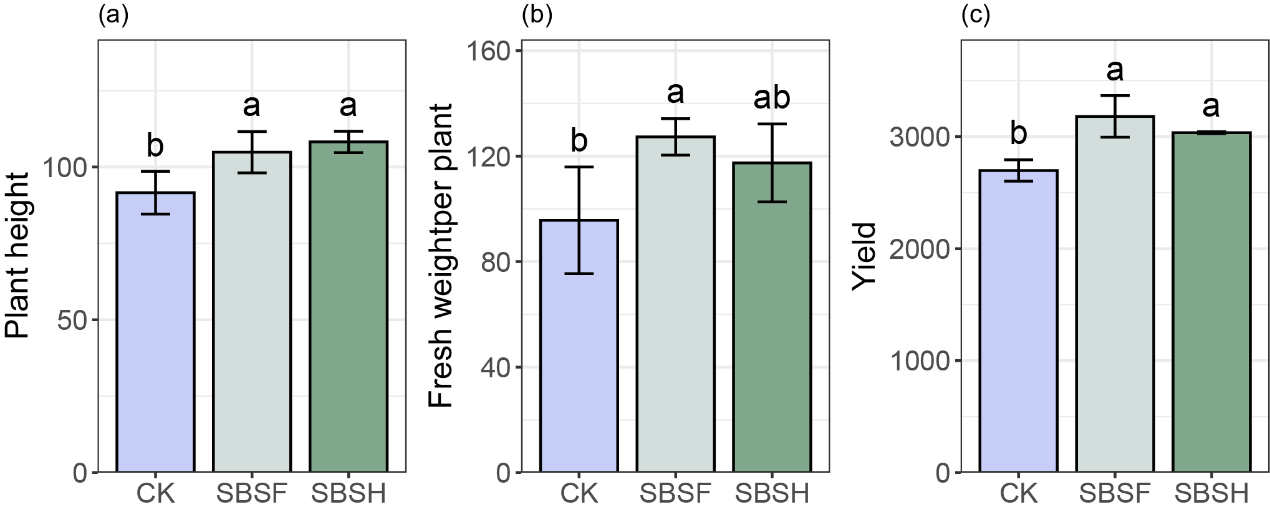


**Supplementary Figure S7.** Changes of soybean growth and yield under different straw returning amounts. Different lowercase letters indicate that there are significant differences between different treatments (*p*＜0.05). CK: no-straw returning; SBSH: half-straw returning; SBSF: full-straw returning.


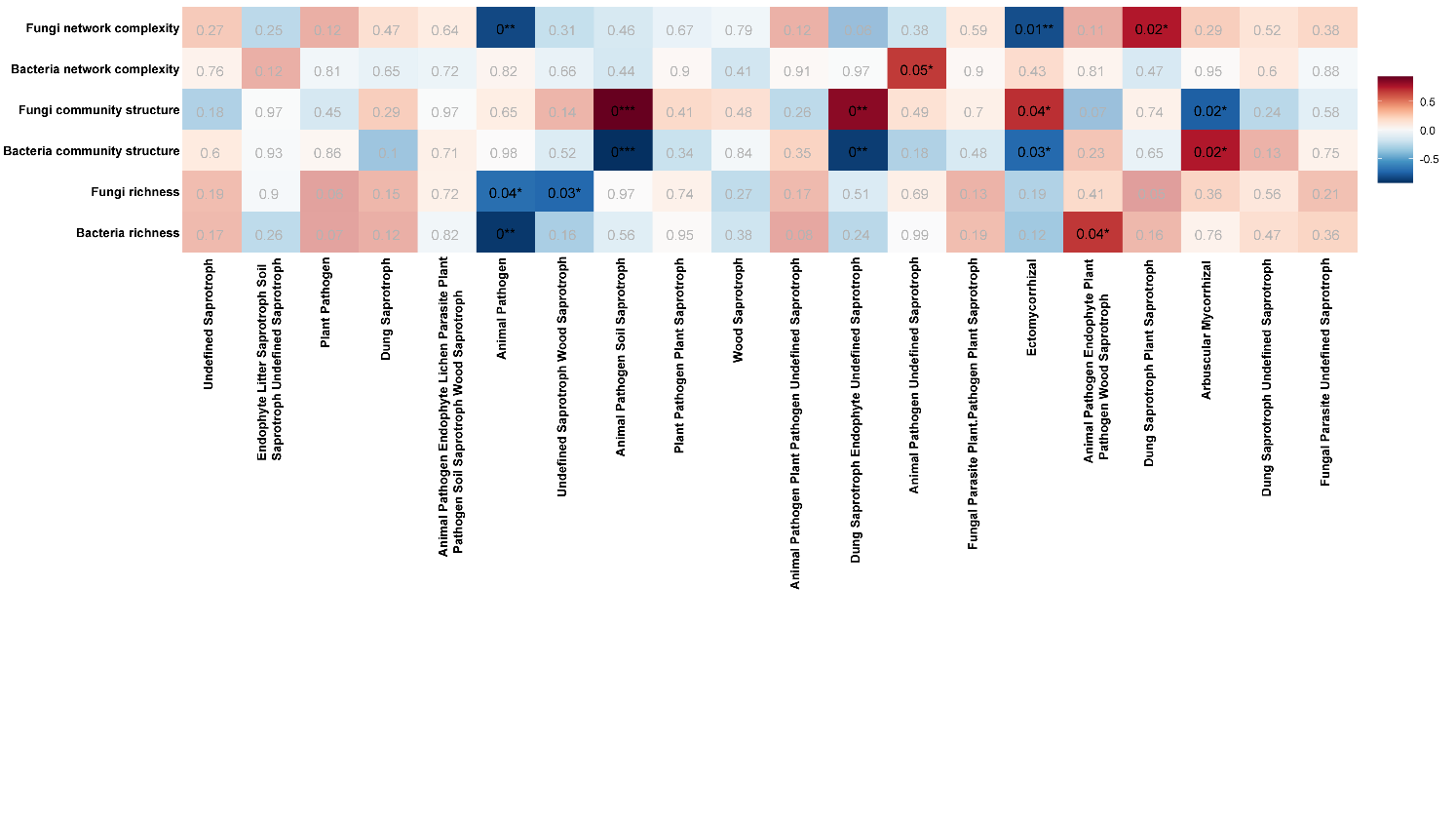


Figure S8 Analysis of the Correlation Between Soil Fungal Functional Traits and Microbial Community Composition

# Supplementary Tables

Supplementary Table S1. Analysis of relative abundance of bacteria at genus level

| Microorganism | CK | SBSF | SBSH |
| --- | --- | --- | --- |
| norank_f__Nitrososphaeraceae | 3.64±1.65b | 8.18±3.29a | 5.98±0.40ab |
| Sphingomonas | 4.63±0.96a | 3.60±0.49a | 4.49±0.54a |
| Candidatus_Udaeobacter | 4.17±1.35a | 4.67±0.98a | 3.45±0.98a |
| norank_o__Gaiellales | 3.60±0.55a | 3.52±0.26a | 3.33±0.15a |
| norank_f__Gemmatimonadaceae | 3.54±0.48a | 3.11±0.35a | 3.32±0.24a |
| norank_o__Vicinamibacterales | 2.65±0.71a | 2.74±0.45a | 2.85±0.63a |
| norank_f__Pyrinomonadaceae | 1.91±0.43a | 2.23±0.43a | 2.08±0.36a |
| Gemmatimonas | 1.93±0.38a | 1.45±0.20a | 1.77±0.20a |
| norank_f__Vicinamibacteraceae | 1.61±0.48a | 1.72±0.29a | 1.68±0.61a |
| Arthrobacter | 1.39±0.36b | 1.39±0.34b | 2.12±0.29a |
| norank_c__MB-A2-108 | 1.23±0.41b | 2.16±0.52a | 1.37±0.32ab |
| norank_o__Terriglobales | 1.40±0.40a | 1.26±0.21a | 1.33±0.24a |
| Rubrobacter | 1.27±0.22a | 1.34±0.17a | 1.36±0.11a |
| norank_c__KD4-96 | 1.23±0.22a | 1.30±0.40a | 1.44±0.25a |
| Gaiella | 1.27±0.27a | 1.31±0.18a | 1.32±0.05a |

Note: The data in the table are expressed as mean ± standard deviation. Different lowercase letters indicate that there are significant differences between different treatments (*p*＜0.05). CK: no-straw returning; SBSH: half-straw returning; SBSF: full-straw returning.

Supplementary Table S2. Analysis of relative abundance of fungi at genus level

| Microorganism | CK | SBSF | SBSH |
| --- | --- | --- | --- |
| Tausonia | 14.32±2.54a | 8.29±1.45b | 14.78±1.55a |
| Schizothecium | 4.55±0.91ab | 3.24±1.15b | 6.00±1.40a |
| Fusarium | 4.34±1.52a | 4.36±0.68a | 4.82±1.64a |
| Pseudombrophila | 4.42±0.57a | 4.66±0.69a | 4.40±0.61a |
| Mortierella | 2.55±0.87b | 4.29±0.47a | 3.15±0.33ab |
| Metacordyceps | 5.34±2.07a | 3.39±2.32ab | 0.78±0.09b |
| Humicola | 8.52±12.48a | 0.37±0.10a | 0.43±0.12a |
| Pseudogymnoascus | 3.39±0.25a | 1.04±0.32b | 3.04±0.25a |
| Linnemannia | 1.22±0.44b | 2.93±0.34a | 2.75±0.65a |
| Plectosphaerella | 0.41±0.22a | 1.52±1.24a | 4.59±4.11a |
| Trichosporiella | 2.15±0.54a | 1.81±0.5a | 1.78±0.74a |
| Septoria | 0.15±0.26a | 2.24±1.99a | 2.92±1.71a |
| Trichocladium | 1.54±0.30a | 1.78±0.36a | 1.43±0.32a |
| Neonectria | 0.89±0.39a | 1.70±0.29a | 1.65±0.55a |
| Uncobasidium | 1.55±1.41a | 1.00±1.73a | 1.63±1.74a |

Note: The data in the table are expressed as mean ± standard deviation. Different lowercase letters indicate that there are significant differences between different treatments (*p*＜0.05). CK: no-straw returning; SBSH: half-straw returning; SBSF: full-straw returning.
